# Personalized Cardio‐Metabolic Responses to an Anti‐Inflammatory Nutrition Intervention in Obese Adolescents: A Randomized Controlled Crossover Trial

**DOI:** 10.1002/mnfr.201701008

**Published:** 2018-05-17

**Authors:** Aoibheann M. McMorrow, Ruth M. Connaughton, Tiago R. Magalhães, Fiona C. McGillicuddy, Maria F. Hughes, David Cheishvili, Melissa J. Morine, Sean Ennis, Marie‐Louise Healy, Edna F. Roche, Richard E. Tremblay, Moshe Szyf, Fiona E. Lithander, Helen M. Roche

**Affiliations:** ^1^ Nutrigenomics Research Group UCD Conway Institute of Biomolecular and Biomedical Research University College Dublin Dublin 4 D04 N2E5 Ireland; ^2^ School of Medicine and Medical Science University College Dublin Dublin 4 D04 N2E5 Ireland; ^3^ Department of Pharmacology and Therapeutics McGill University Medical School Montreal QC QC H3G 2M1 Canada; ^4^ The Microsoft Research–University of Trento Centre for Computational and Systems Biology Rovereto 38068 Italy; ^5^ Department of Endocrinology St. James's Hospital Dublin 8 D08 C9X2 Ireland; ^6^ Department of Pediatrics Trinity College Dublin The University of Dublin Dublin 2 D02 W272 Ireland; ^7^ The National Children's Hospital Tallaght, Dublin 24 D24 AP52 Ireland; ^8^ St‐Justine Hospital Research Centre Montreal QC QC H3T 1C5 Canada; ^9^ Departments of Pediatrics and Psychology University of Montreal Montreal QC QC H4A 3J1 Canada; ^10^ School of Public Health Physiotherapy and Sports Science University College Dublin Dublin 4 D04 N2E5 Ireland; ^11^ NIHR Bristol Biomedical Research Centre (Nutrition Theme) at University Hospitals Bristol NHS Foundation Trust and the University of Bristol BS1 2LY United Kingdom

**Keywords:** adolescents, inflammation, insulin resistance, pediatric obesity, personalized nutrition

## Abstract

**Scope:**

Chronic inflammation and hypoadiponectinemia are characteristics of obesity‐induced insulin resistance (IR). The effect of an anti‐inflammatory nutrition supplement (AINS) on IR and adiponectin biology in overweight adolescents was investigated. The secondary objective was to examine the extent to which individuals’ biomarker profiles, derived from baseline phenotypes, predicted response or not to the AINS. Additionally, the impact of DNA methylation on intervention efficacy was assessed.

**Methods and results:**

Seventy overweight adolescents (13–18 years) were recruited to this randomized controlled crossover trial. Participants received an AINS (long chain n‐3 PUFA, vitamin C, α‐tocopherol, green tea extract, and lycopene) and placebo for 8 weeks each. Homeostatic model assessment (HOMA)‐IR, adiponectin, inflammatory profiles, and DNA methylation were assessed. HOMA‐IR was unchanged in the total cohort. High‐molecular‐weight (HMW) adiponectin was maintained following the AINS while it decreased over time following the placebo intervention. HOMA‐IR decreased in 40% of subjects (responders) following the AINS. Responders’ pretreatment phenotype was characterized by higher HOMA‐IR, total and LDL cholesterol, but similar BMI in comparison to nonresponders. HMW adiponectin response to the AINS was associated with bidirectional modulation of adipogenic gene methylation.

**Conclusion:**

The AINS modulated adiponectin biology, an early predictor of type 2 diabetes risk, was associated with bidirectional modulation of adipogenic gene methylation in weight‐stable overweight adolescents. HOMA‐IR decreased in a sub‐cohort of adolescents with an adverse metabolic phenotype. Thus, suggesting that more stratified or personalized nutrition approaches may enhance efficacy of dietary interventions.

## 1 Introduction

Pediatric obesity is of major public health concern^1^; one in three 11‐year‐old children in Europe are overweight or obese.^2^ Though the incidence of type 2 diabetes (T2D) in obese children and adolescents is rare,^3^ it is likely that the progressive decline in insulin sensitivity initiates in youth.^4^ Despite obesity, however, some adolescents maintain insulin sensitivity.^5^, ^6^ Relative to insulin‐resistant obese adolescents, insulin‐sensitive obese adolescents demonstrate higher circulating adiponectin, an anti‐inflammatory adipocytokine.^7^ Whilst glucose, insulin, and HbA1c concentrations are important diagnostic biomarkers of T2D, adiponectin, and to a greater extent its high‐molecular‐weight (HMW) oligomeric complex, are also early and sensitive biochemical predictors of future T2D.^8^, ^9^, ^10^, ^11^, ^12^, ^13^ Indeed, in overweight and obese adolescents, hypoadiponectinemia was demonstrated to precede cardio‐metabolic dysregulation by 6 years.^14^ Furthermore, despite normal fasting glucose and HbA1c levels, obese adolescents with insulin‐resistance as measured by hyperinsulinemic‐euglycemic clamp demonstrated deranged serum adiponectin.^11^

Approximately 70% of T2D may be prevented or delayed through lifestyle intervention.^15^ Besides positive energy balance, dietary fat quality may be a potential determinant of IR and T2D.^16^, ^17^ Saturated fatty acid (SFA)–rich high fat diets (HFD) induce IR, adipose tissue inflammation, hypertrophy, and hypoxia.^18^, ^19^ In contrast, a HFD supplemented with MUFA or long chain (LC) n‐3 PUFA is associated with adipose hyperplasia and attenuated inflammation.^18^, ^20^ The impact of dietary fatty acid modification on insulin sensitivity and adiponectin in overweight adolescents however is poorly understood.^21^

In contrast to single‐nutrient approaches, it has been proposed that a combination of anti‐inflammatory nutrients may increase intervention efficacy.^22^, ^23^ One such approach, a Mediterranean style diet, decreased fasting plasma glucose, triacylglycerol (TAG), and BMI in obese adolescents.^24^ However, the therapeutic potential of a combined anti‐inflammatory nutrition intervention in a weight‐stable obese pediatric cohort is unknown.

Responsiveness to nutrition intervention may be partially attributable to baseline phenotype and genetic background.^25^ Furthermore, the epigenome, the interface between genetics and the environment, may modulate response to dietary intervention as previously demonstrated.^26^ Personalized nutrition presents an opportunity to optimize intervention efficacy wherein the paradigm recognizes interindividual heterogeneity.^27^ In terms of personalized nutrition, we refer to the opportunity to more effectively assess health status versus disease risk in susceptible subpopulations. Whilst not an individualized *n* = 1 approach per se, identifying or stratifying subgroups may (or may not) improve the potential efficacy of nutritional interventions. Childhood obesity is a substantial health and economic burden,^28^, ^29^ therefore personalized diagnoses and treatment of obesity and its metabolic complications is a potential approach that requires investigation for substantiation.

This study addressed the hypothesis that a combination of anti‐inflammatory nutrients modulates IR and related secondary outcomes such as adiponectin, lipid concentrations, and inflammatory markers in weight‐stable overweight and obese adolescents. Furthermore, the impact of baseline phenotype and changes in DNA methylation on intervention efficacy was explored.

## 2 Experimental Section

### 2.1 Study Design

This randomized double‐blind, placebo‐controlled, crossover trial, named the Teen Nutrition Study, investigated the effect of an anti‐inflammatory nutrition supplement (AINS) versus placebo in weight‐stable overweight and obese adolescents. Participants and their parent(s)/guardian(s) attended one of the three study sites for data collection; 1) the Institute of Food and Health, University College Dublin, 2) the Trinity Centre for Health Sciences, Trinity College Dublin, the University of Dublin, or 3) the National Children's Hospital, Dublin, Ireland. Data were collected by the same research investigators (A.M.M., R.M.C.) at all study sites. The study protocol was approved by the Joint Research Ethics Committee of St. James's Hospital and the Adelaide and Meath Hospital, incorporating the National Children's Hospital, Dublin, Ireland. This trial was registered with ClinicalTrials.gov (NCT01665742).

### 2.2 Participants

Between January 4, 2012 and April 30, 2013, 70 overweight and obese adolescents, aged 13–18 years, were recruited from pediatric weight management outpatient clinics and the general public. Overweight and obesity were defined as a BMI ≥91st and ≥98th percentiles on United Kingdom growth reference charts,^30^ respectively. Exclusion criteria are presented in the online supporting material. Information relating to the purpose, procedures, potential benefits, and risks of the study was provided before informed written assent/consent was obtained from participants and their parent(s)/guardian(s), respectively.

### 2.3 Randomization and Masking

Participants were randomized to receive either the AINS or placebo for the initial treatment period. Randomization was conducted in a ratio of 1:1 and was stratified according to age, sex and baseline BMI using the MINIM randomization program.^31^ The research dietitian (A.M.M.), participants, parents/guardians, intervention providers, and outcome analyzers were blinded.

### 2.4 Intervention

Participants received either the AINS or placebo daily; with healthy eating advice for a period of 8 weeks. Nutrition education was provided at the beginning of each treatment period to encourage weight maintenance. Weight management education was based on the Traffic Light Diet.^32^ Identical advice was provided regardless of treatment group allocation. After a 6 weeks washout, the alternate treatment was received daily for 8 weeks. Anthropometric data and fasting blood samples were collected pre‐ and post‐intervention arms.

The AINS comprised LC n‐3 PUFA (1000 mg eicosapentaenoic acid [EPA] and 1000 mg docosahexaenoic acid [DHA]), 567 mg vitamin C, 390 mg α‐tocopherol, 416 mg green tea extract (45% epigallocatechin‐3‐gallate), and 16.5 mg lycopene. These nutrients were delivered via two study products; 1 × 200 mL carton of fruit juice fortified with emulsified fish oil (Smartfish, Oslo, Norway) and 4× film coated tablets containing vitamin C, α‐tocopherol, green tea extract, and lycopene (Best Formulations, CA, USA). Matching placebo treatment comprised an iso‐energetic fruit juice fortified with high oleic sunflower oil and 4× film coated tablets containing microcrystalline cellulose. Apart from the fatty acid content, the placebo fruit juice was identical to its active counterpart in nutrient composition. The active and placebo treatments matched for size, shape, color, and flavor, labeled A or B. Participants were instructed to consume supplements daily, with breakfast.

### 2.5 Outcomes

The primary outcome was HOMA‐IR.^33^ Secondary outcomes were plasma adiponectin, lipid and lipoprotein concentrations, inflammatory profile, body composition, and DNA methylation. All outcomes were assessed after an overnight fast.

Anthropometric measurements were conducted using calibrated equipment. Blood for insulin, glucose, lipid, and inflammatory profiles was collected and processed as previously described.^31^ Peripheral blood mononuclear cells (PBMC) were isolated from whole blood^34^ and treated with LPS (10 μg mL^−1^) for 24 hours. Cytokine secretion from unstimulated and LPS‐stimulated PBMC was assessed for subacute inflammatory phenotyping indicative of adipose tissue inflammation.^34^ Supernatants were collected and stored at −80 °C until analyzed. PBMC RNA isolation is described elsewhere.^18^

ELISA determined total and HMW adiponectin, soluble CD163 (sCD163), IL‐6, tumor necrosis factor‐α (TNF‐α; R&D Systems, UK), insulin (Mercodia, Sweden), complement component C3 (AssayPro, MO, USA), and fetuin‐A (BioVendor, Czech Republic). Plasma glucose, total cholesterol, HDL cholesterol, TAG, and apolipoprotein A1 (ApoA1) were measured using RX Daytona, as described previously.^35^ Plasma nonesterified fatty acid (NEFA) concentration was measured by an enzymatic assay kit (WAKO Diagnostics, USA). Intra‐ and inter‐assay CVs were <10% for all analyses.

DNA was extracted from buffy coats using the QIAamp DNA Blood Mini Kit (Qiagen Ltd, Crawley, UK). DNA methylation analysis is described in the online supporting material. Briefly, DNA methylation was analyzed in *n* = 55 samples by the McGill University and Génome Québec Innovation Centre (Montreal, Quebec, Canada) using the Infinium HumanMethylation450 BeadChip assay (Illumina, San Diego, CA, USA). Biologically relevant microarray results were technically validated using Sequenom EpiTYPER in a sub‐cohort of participants (*n* = 22) who demonstrated DNA methylation changes of greatest magnitude in both directions.

### 2.6 Dietary Intervention Compliance Assessment

Plasma cholesteryl ester fatty acid composition, α‐tocopherol and lycopene were determined pre‐ and post‐intervention arms to assess compliance, as described previously.^31^, ^36^, ^37^, ^38^

### 2.7 Adverse Events

Adverse events (AEs) were recorded and examined by the pediatric physician (KJ). One AE, a potential intolerance to whey within the fortified juice, occurred. The participant discontinued the trial. No serious adverse events occurred.

### 2.8 Statistical Analyses

Sample size calculations were based on the hypothesized decrease in HOMA‐IR. The aim was to enroll 70 participants to give 80% power (*p* < 0.05) to detect a 15% change in HOMA‐IR. Sample size was adjusted for 30% attrition and an anticipated 50% nonresponse rate, to provide sufficient power for the analysis of intervention responsiveness. Statistical analyses were completed using SPSS Statistics (Version 20; IBM, IL, USA). Intention‐to‐treat analysis was used to investigate the effect of the intervention on HOMA‐IR, total and HMW‐adiponectin, and BMI. Participants who discontinued the study were younger and did not differ from adolescents who completed the intervention with respect to gender distribution or age‐ and gender‐normalized weight, height, or body composition (Table S1, Supporting Information). All data were examined for Gaussian distribution, and non‐normally distributed data were transformed. Comparisons between AINS and placebo were made using paired samples *t*‐tests between delta AINS and delta placebo values. Independent samples *t*‐tests examined differences in baseline characteristics of participants who completed the trial versus those who discontinued. To explore heterogeneity in responsiveness to the intervention, “responders” were defined as those demonstrating a significant improvement (≥10% reduction in HOMA‐IR) following the AINS while “nonresponders” demonstrated <10% decrease in HOMA‐IR. A 10% response threshold was based on longitudinal research illustrating a mean improvement of approximately 8% in HOMA‐IR in adolescents who transitioned from an overweight BMI to a healthy BMI.^39^ Analyses were repeated in this sub‐cohort to quantify the intervention effect in responsive adolescents. To validate findings, multivariate stepwise regression was the unsupervised statistical approach. Independent samples *t*‐tests examined differences in baseline characteristics between responders versus nonresponders. For biomarkers showing significant differences between responders and nonresponders, optimal cutpoints with their percentage accuracy were derived from ROC analysis (GraphPad Prism, V5, CA, USA). Epigenetic data were processed using the Bioconductor Minfi package in R,^40^ as described in the online supporting material.

Results are presented as mean (SEM) in figures and as mean (SD) in text and tables.

## 3 Results

**Figure 1:**
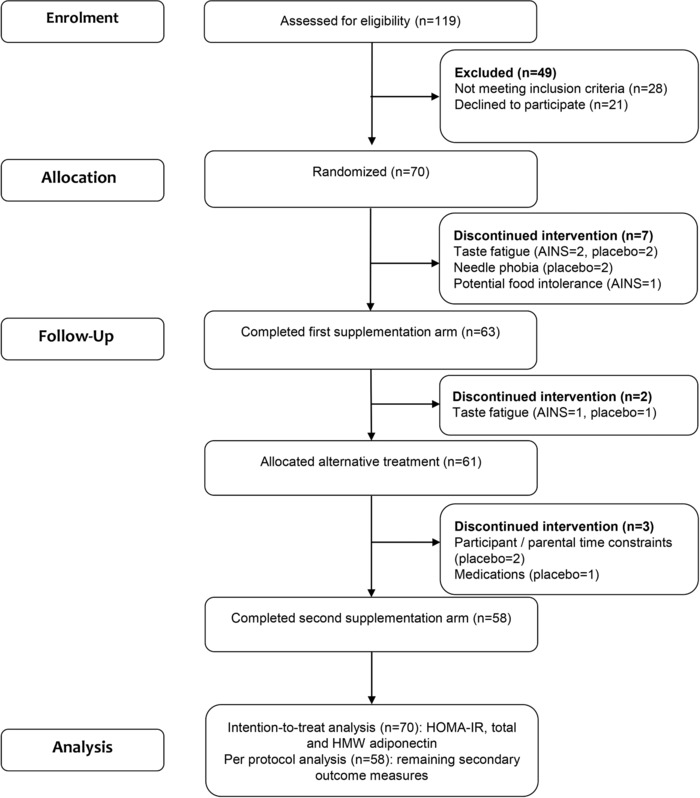
Trial profile. AINS, anti‐inflammatory nutrition supplement; HOMA‐IR, homeostatic model assessment‐insulin resistance.

The consort diagram illustrates that 70 adolescents were randomly assigned to receive the AINS or placebo for 8 weeks (**Figure** 1). Fifty‐eight (83%) participants completed the intervention, 12 (17%) discontinued the trial, the majority of whom dropped out after their initial baseline assessment, details of which are presented in Table S1, Supporting Information. Twenty‐seven percent of participants were overweight, 73% were obese, and 22% had severe obesity (BMI SDS > 3.5).

Pre‐ and post‐intervention anthropometry, insulin, glucose, lipid and inflammatory marker concentrations in completers are presented in **Table** 1.

**Table 1:** Anthropometry, insulin, glucose, lipid, and inflammatory biomarker concentrations pre‐ and post‐intervention following the AINS versus placebo intervention for 8 weeks (completers *n* = 58)

	AINS		Placebo		*p*‐value
	Pre	Post	Pre	Post	Intervention
Weight [kg]	92.78 (22.83)	92.60 (23.00)	92.77 (22.78)	92.63 (22.22)	0.94
BMI [kg m^−2^]	31.7 (6.3)	31.5 (6.3)	31.7 (6.3)	31.5 (6.1)	0.95
Glucose and insulin homeostasis					
Glucose [mmol L^−1^]	5.19 (0.38)	5.15 (0.40)	5.20 (0.41)	5.20 (0.36)	0.48
Insulin [mU L^−1^]	11.59 (6.72)	10.89 (5.77)	11.50 (7.07)	10.84 (5.39)	0.97
HOMA‐IR	2.61 (1.64)	2.43 (1.44)	2.58 (1.72)	2.43 (1.30)	0.99
QUICKI	0.34 (0.03)	0.34 (0.02)	0.34 (0.02)	0.34 (0.02)	0.60
Lipids and lipoproteins					
TAG [mmol L^−1^]	0.97 (0.47)	0.95 (0.47)	0.99 (0.40)	1.00 (0.47)	0.59
Fetuin A [μg mL^−1^]	573.55 (333.35)	606.98 (399.38)	590.17 (370.71)	616.31 (343.39)	0.28
NEFA [mEq L^−1^]	0.51 (0.19)	0.60 (0.27)	0.52 (0.23)	0.50 (0.25)	0.99
Total cholesterol [mmol L^−1^]	3.80 (0.71)	3.88 (0.66)	3.89 (0.70)	3.78 (0.65)	0.06
HDL cholesterol [mmol L^−1^]	1.22 (0.28)	1.23 (0.28)	1.25 (0.31)	1.19 (0.25)	0.57
LDL cholesterol [mmol L^−1^]	2.14 (0.53)	2.22 (0.50)	2.19 (0.55)	2.13 (0.51)	0.09
LDL:HDL ratio	1.84 (0.55)	1.89 (0.57)	1.88 (0.83)	1.87 (0.68)	0.87
ApoA1 [mg dL^−1^]	111 (18)	109 (19)	115 (20)	111 (16)	0.36
Inflammatory profile					
Plasma total adiponectin [μg mL^−1^]	7.57 (3.99)	7.70 (4.47)	8.09 (4.59)	7.55 (3.70)	0.79
Plasma HMW adiponectin [μg mL^−1^]	3.78 (2.96)	4.01 (3.26)	3.77 (2.77)	3.28 (2.41)	0.004
Plasma leptin [ng mL^−1^]	30.2 (23.6)	29.0 (21.8)	24.7 (26.7)	32.1 (25.2)	0.65
Plasma FABP4 [ng mL^−1^]	26.6 (23.0)	25.9 (17.2)	23.7 (18.7)	27.9 (20.63	0.43
Serum sCD163 [ng mL^−1^]	690.1 (303.5)	701.6 (314.4)	693.8 (297.3)	648.1 (283.7)	0.14
Plasma complement C3 [mg mL^−1^]	0.6 (0.3)	0.5 (0.3)	0.7 (0.4)	0.6 (0.4)	0.47
Plasma IL‐6 [pg mL^−1^]	2.4 (0.9)	2.5 (0.9)	2.4 (0.7)	2.5(1.0)	0.97
PBMC IL‐6 unstimulated [pg mL^−1^]	120.1 (216.8)	94.2 (200.7)	70.2 (124.0)	83.2 (178.2)	0.24
PBMC IL‐6 LPS stimulated [pg mL^−1^]	10879.5 (8076.2)	10383.2 (7290.6)	10848.7 (8065.9)	11255.0 (7590.5)	0.31
Plasma TNF‐α [pg mL^−1^]	10.4 (1.3)	10.7 (1.6)	10.4 (1.2)	11.0 (1.4)	0.18
PBMC TNF‐α unstimulated [pg mL^−1^]	41.7 (127.5)	24.9 (101.3)	23.0 (99.9)	12.9 (17.8)	0.36
PBMC TNF‐α LPS‐stimulated [pg mL^−1^]	291.3 (198.8)	286.4 (217.9)	345.7 (425.0)	332.0 (384.5)	0.78

Data are presented as mean (SD); Data were analyzed in completers (*n* = 58) using paired samples *t*‐tests between delta supplement and delta placebo values. For BMI, HOMA‐IR, total, and HMW adiponectin, data were analyzed in all *n* = 70 participants who commenced the trial using intention‐to‐treat analysis; ApoA1, apolipoprotein A1; FABP4, fatty acid binding protein 4; HOMA‐IR, homeostatic model assessment‐insulin resistance; NEFA, nonesterified fatty acids; PBMC, peripheral blood mononuclear cells; QUICKI, quantitative insulin sensitivity check index; TAG, triacylglycerol; TNF‐α, tumor necrosis factor‐α; HMW, high molecular weight.

### 3.1 The AINS Modulated Adiponectin Biology But Not Insulin Resistance

**Figure 2:**
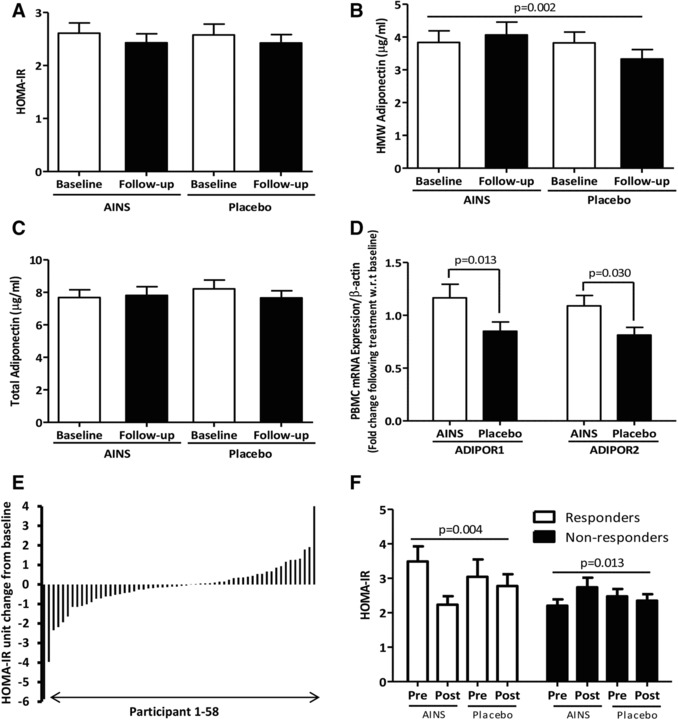
Changes in HOMA‐IR and adiponectin following the AINS versus placebo. A) HOMA‐IR, B) high‐molecular‐weight adiponectin, C) total adiponectin, and D) adiponectin receptor mRNA expression following the AINS relative to placebo (intention‐to‐treat analysis, *n* = 70). E) Heterogeneity in response to the AINS with respect to HOMA‐IR in completers (*n* = 58). F) Change in HOMA‐IR following the AINS relative to placebo in responders and nonresponders (*n* = 58). Data are presented as mean (SEM). Data were analyzed using paired samples *t*‐tests between delta supplement and delta placebo values. ADIPOR1, adiponectin receptor 1; ADIPOR2, adiponectin receptor 2; AINS, anti‐inflammatory nutrition supplement; HMW, high‐molecular‐weight; HOMA‐IR, homeostatic model assessment‐insulin resistance; PBMC, peripheral blood mononuclear cells.

The AINS did not change HOMA‐IR in the total cohort (**Figure** 2A), despite significant modulation of adiponectin biology. HMW adiponectin was maintained following the AINS while it decreased over time following the placebo intervention (Figure 2B). Total adiponectin (Figure 2C) and BMI (AINS 31.49(6.31) kg m^−2^ vs placebo 31.50(6.08) kg m^−2^, *p* = 0.951, *n* = 70) remained stable. Adiponectin receptor ADIPOR1 and ADIPOR2 mRNA levels were significantly higher in PBMC following the AINS, relative to placebo (Figure 2D). There was no significant effect of the intervention on plasma glucose or serum insulin (Table 1).

### 3.2 Insulin Resistance Improved in a Sub‐Cohort of Adolescents

A key objective was to explore interindividual HOMA‐IR responses to the AINS. We observed considerable heterogeneity in intervention responsiveness (mean HOMA‐IR change following AINS in completers; −0.18 units [95% CI; −0.54, 0.18]; Figure 2E). Stratification of the cohort according to the direction and magnitude of response revealed that 23 (40%) adolescents responded favorably to the AINS, demonstrating a minimum of a 10% improvement in HOMA‐IR following the active treatment (responder‐by‐intervention effect *p* = 0.001). The reduction in HOMA‐IR following the AINS was significantly greater than placebo in responders (*p* = 0.004; Figure 2F) attributable to decreased insulin rather than glucose concentration (Table S2, Supporting Information). By contrast, HOMA‐IR increased significantly in nonresponders (Figure 2F). Importantly, objective biomarkers of compliance increased significantly following the active compared to placebo treatment in the total cohort (Table S3, Supporting Information), and no differences in compliance were observed between responders and nonresponders (Figure S1, Supporting Information).

### 3.3 Baseline Phenotype Predicted Insulin Resistance Response to Intervention

The baseline metabolic phenotype of responders was insulin resistant and dyslipidemic with higher insulin, HOMA‐IR, HOMA‐β, total cholesterol, LDL cholesterol, and lower QUICKI (**Table** 2). In contrast, gender, age, BMI, and body composition, were not different between responders and nonresponders (Table 2). Multiple regression analyses further validated baseline metabolic phenotype as a predictor of HOMA‐IR response to the AINS. Baseline HOMA‐IR (*β* = −0.659, 95% CI, −1.052, −0.266, *p* < 0.001), sCD163 (*β* = 0.292, 95% CI, 0.218, 0.366, *p* = 0.003), and LDL:HDL cholesterol ratio (*β* = 0.337, 95% CI, 0.237, 0.437, *p* = 0.001) all independently predicted HOMA‐IR response. Additionally, changes in sCD163 (*β* = 0.556, 95% CI, 0.281, 0.831, *p* < 0.001) following the AINS tracked HOMA‐IR changes (final regression model; *R* ^2^ = 0.729, *p* < 0.001). By contrast, compliance, baseline BMI and BMI change did not predict HOMA‐IR response (*p* > 0.05 for all, data not shown). In retrospective ROC analysis, baseline HOMA‐IR level over 1.83 was 45.7% sensitive and 95.6% specific at differentiating responders from nonresponders to the AINS (Table S4, Supporting Information), which was marginally less predictive than baseline insulin (8.2 mU L^−1^, 51.4% sensitive/95.5% specific).

**Table 2:** Baseline characteristics of responders (*n* = 23) and nonresponders (*n* = 35) to the anti‐inflammatory nutrition supplement

	Responders	Nonresponders	*p*‐value
Male:female [%]	30:70	46:54	0.28
Age [years]	16.1 (1.9)	16.1 (1.5)	0.91
Body composition			
Weight [kg]	94.77 (24.55)	91.46 (21.90)	0.60
BMI [kg m^−2^]	33.0 (7.0)	31.1 (6.2)	0.27
Waist circumference [cm]	106.74 (13.98)	103.85 (14.29)	0.42
Fat mass [kg]	37.64 (15.36)	33.98 (16.63)	0.22
Body fat [%]	38.84 (6.88)	35.94 (10.29)	0.24
Fat mass index [kg m^−2^]	13.13 (5.09)	11.67 (5.55)	0.16
Fat free mass [kg]	57.12 (12.70)	57.27 (11.94)	0.22
Fat free mass index [kg m^−2^]	19.83 (2.93)	19.45 (2.44)	0.62
Fat:fat free mass ratio	0.66 (0.21)	0.61 (0.30)	0.20
Metabolic phenotype			
Glucose [mmol L^−1^]	5.22 (0.36)	5.17 (0.39)	0.60
Insulin [mU L^−1^]	14.86 (8.43)	9.44 (4.20)	0.001
HOMA‐IR	3.49 (2.10)	2.21 (1.07)	0.001
HOMA‐β [%]	172.82 (87.67)	112.76 (40.24)	<0.001
QUICKI	0.32 (0.02)	0.35 (0.03)	0.001
TAG [mmol L^−1^]	1.01 (0.49)	0.95 (0.45)	0.66
Fetuin A [μg mL^−1^]	504.78 (309.34)	616.78 (344.86)	0.22
NEFA [mEq L^−1^]	0.47 (0.17)	0.58 (0.23)	0.13
Total cholesterol [mmol L^−1^]	4.05 (0.74)	3.64 (0.64)	0.03
HDL cholesterol [mmol L^−1^]	1.25 (0.22)	1.19 (0.32)	0.41
LDL cholesterol [mmol L^−1^]	2.34 (0.56)	2.02 (0.48)	0.02
LDL:HDL ratio	1.92 (0.54)	1.78 (0.55)	0.35
ApoA1 [mg dL^−1^]	114.11 (16.12)	109.53 (19.43)	0.35
Inflammatory profile			
Total adiponectin [μg mL^−1^]	7.4 (4.8)	7.4 (3.5)	0.93
HMW adiponectin [μg mL^−1^]	3.7 (2.8)	3.7 (3.2)	0.64
Leptin [ng mL^−1^]	39.1 (25.8)	27.7 (22.2)	0.08
FABP4 [ng mL^−1^]	25.6 (16.1)	22.2 (20.1)	0.50
sCD163 [ng mL^−1^]	702.0 (293.4)	662.5 (330.3)	0.22
Complement C3 [mg mL^−1^]	0.7 (0.4)	0.5 (0.2)	0.61
Plasma IL‐6 [pg mL^−1^]	2.4 (0.6)	2.4 (1.1)	0.42
PBMC IL‐6 unstimulated [pg mL^−1^]	166.9 (251.6)	90.0 (190.3)	0.29
PBMC IL‐6 LPS stimulated [pg mL^−1^]	9287.1 (8662.7)	11903.8 (7660.5)	0.29
Plasma TNF‐α [pg mL^−1^]	10.4 (1.5)	10.4 (1.3)	0.99
PBMC TNF‐α unstimulated [pg mL^−1^]	78.9 (194.8)	16.5 (27.5)	0.84
PBMC TNF‐α LPS stimulated [pg mL^−1^]	284.2 (242.0)	295.9 (170.0)	0.79

Data are presented as mean (SD); Data were analyzed by independent samples *t*‐test between responders and nonresponders; BMI (kg m^−2^) = weight (kg) / height (m^2^); fat mass index (kg m^−2^) = fat mass (kg) / height (m^2^); fat free mass index (kg m^−2^) = fat free mass (kg) / height (m^2^); ApoA1, apolipoprotein A1; FABP4, fatty acid binding protein 4; HOMA‐IR, homeostatic model assessment‐insulin resistance; HOMA‐β, homeostatic model assessment‐β‐cell function; NEFA, nonesterified fatty acids; PBMC, peripheral blood mononuclear cells; QUICKI, quantitative insulin sensitivity check index; sCD163, soluble CD1163; TAG, triacylglycerol; TNF‐α, tumor necrosis factor‐α.

### 3.4 HMW Adiponectin Response was Associated with Modulated Methylation of Adipogenic Genes

**Figure 3:**
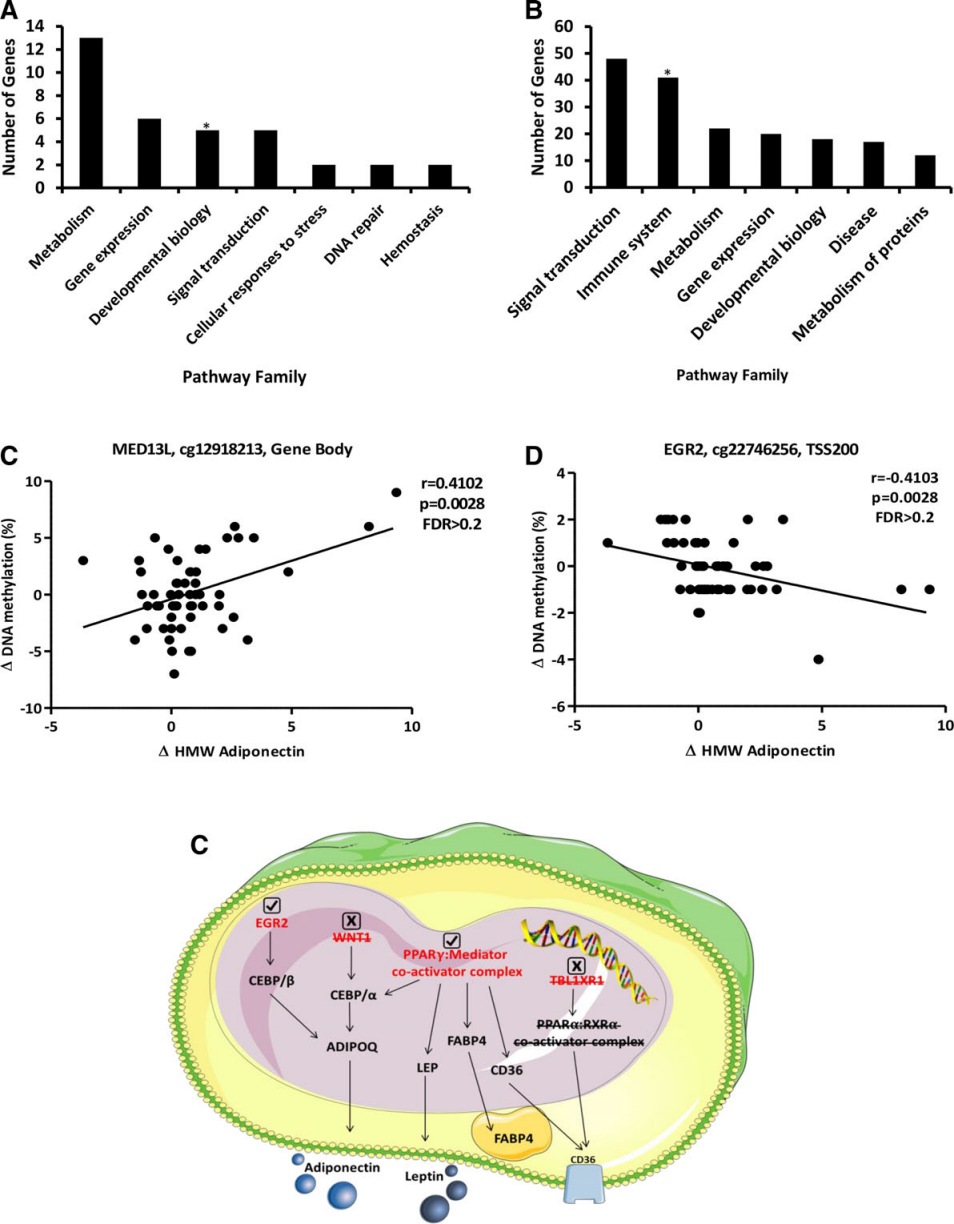
Modulation of DNA methylation in response to the AINS (*n* = 55). Reactome pathway families which demonstrated A) positive and B) inverse associations between DNA methylation and HMW adiponectin changes in response to the AINS. *indicates significant pathway enrichment (*p* ≤ 0.05), as determined by right‐tailed Fisher's exact test. Changes in the methylation status of CpG loci located on C) MED13L and D) EGR2 in relation to HMW adiponectin response to the AINS as determined by Illumina (completers only). E) Schematic demonstrating adipogenic genes (red) that contained CpG loci demonstrating significant associations between methylation status and HMW adiponectin response as assessed by Illumina analysis. Downstream targets are marked in black. Genes in which associations were verified by EpiTYPER are represented by a check mark. Genes in which associations were not confirmed upon technical validation are represented by an “x” mark. HMW, high‐molecular‐weight; FDR, false discovery rate.

We investigated whether changes in DNA methylation in response to the AINS were related to HMW adiponectin response. We observed significant associations between the changes in HMW adiponectin and the methylation status of 487 CpG loci following the AINS (*p* < 0.05 and absolute correlation co‐efficient >0.4 for all, Table S5, Supporting Information). Reactome pathway analyses demonstrated that positively associated CpG loci were predominantly located on metabolic genes as well as on genes implicated in developmental biology and gene expression (**Figure** 3A). In contrast, inversely associated CpG loci were on or near genes largely associated with signal transduction and the immune system (Figure 3B). Furthermore, within the context of developmental biology pathways, transcriptional regulation of white adipose tissue differentiation demonstrated significant enrichment (*p* = 0.03), as did cytokine signaling in the immune system (*p* = 0.001). Figures 3C,D (and Figure S2, Supporting Information) show the methylation status of CpG loci from the five adipose tissue differentiation genes which demonstrated significant associations with HMW adiponectin response (EGR2, WNT1, MED4, MED13L, and TBL1XR1).

Statistically significant relationships from Pearson correlation analysis and pathway enrichment analysis did not persist after correction for multiple comparisons (Benjamini–Hochberg false discovery rate [FDR] = 1 for all). However, given the potential biological relevance of these adipogenic gene methylation changes, we sought to validate the findings using an alternate platform. EpiTYPER analysis confirmed statistically significant associations between the changes in HMW adiponectin and the methylation status of CpG loci located on EGR2 and MED4 (Figure 3E and Table S6, Supporting Information). Conversely, changes in the methylation status of CpG loci on WNT1, MED13L, and TBL1XR1 showed no significant associations with HMW adiponectin response upon validation (Figure 3E and Table S6, Supporting Information). We also sought to validate the methylation status of CpG loci on INSR and KLF14 given that both genes have been implicated in T2D risk according to genome‐wide association studies.^41^ Similarly, Illumina analysis demonstrated associations with HMW adiponectin response that did not persist after correction for multiple testing (Figure S5, Supporting Information). Despite nonsignificant FDR values however, EpiTYPER analysis verified statistically significant associations between HMW adiponectin response and changes in the methylation status of both INSR and KLF14 (Table S6, Supporting Information).

## 4 Discussion

This study demonstrated that whilst an AINS did not affect HOMA‐IR in the total cohort, it maintained HMW adiponectin concentration, coincident with upregulated adiponectin receptor ADIPOR1 and ADIPOR2 mRNA expression in weight‐stable overweight and obese adolescents. Furthermore, modulation of the CpG methylation status of adipogenic genes was related to the HMW adiponectin response. Interestingly from a personalized nutrition perspective, HOMA‐IR decreased in a sub‐cohort of participants considered to be responders, despite equivalent compliance to nonresponders. Although baseline BMI was comparable between groups, pretreatment responders were more insulin resistant, with higher total and LDL cholesterol concentrations. We provide proof‐of‐principle evidence that intervention response varies by baseline metabolic phenotype, wherein an AINS may have the potential to attenuate IR in high‐risk overweight and obese adolescents.

Adiponectin is an insulin‐sensitizing adipocytokine that preserves hepatic insulin sensitivity via a number of mechanisms including increased AMP‐activated protein kinase phosphorylation and reduced expression of gluconeogenic enzymes.^42^, ^43^ HMW adiponectin is a greater predictor of T2D risk than total adiponectin,^8^ potentially attributable to increased binding affinity to adiponectin receptors.^44^ While several RCTs have examined the impact of LC n‐3 PUFA on adiponectin,^21^, ^45^ few studies have examined the effect of anti‐inflammatory nutritional combinations. Neale et al. (2013) demonstrated that six oily fish servings per week for 4 weeks increased HMW adiponectin but an equivalent dose of LC n‐3 PUFA as a supplement had no effect, in weight‐stable overweight adults.^46^ Similarly, LC n‐3 PUFA supplementation did not alter HMW adiponectin in obese adolescents.^47^ In this study, LC n‐3 PUFA supplementation in combination with additional anti‐inflammatory nutrients, maintained circulating HMW adiponectin in overweight and obese adolescents.

Adiponectin mediates its insulin‐sensitizing effects via its receptors ADIPOR1 and ADIPOR2, the expression of which are downregulated in obesity.^48^ Using PBMC as a surrogate for adipose tissue,^34^ the AINS increased ADIPOR1 and ADIPOR2 mRNA expression relative to placebo treatment. The maintenance of HMW adiponectin concentration, concurrent with increased adiponectin receptor mRNA expression, may attenuate future T2D risk in the absence of weight loss.

Although DNA methylation is heritable, the epigenome is flexible and responsive to environmental stimuli.^49^ Here we demonstrated that altered methylation of genes implicated in adipogenesis and T2D risk was associated with HMW adiponectin response to the AINS. Whilst we acknowledge that correction for multiple testing is a critical component of high dimensional data analysis, the results of this analysis call into question the importance of statistical significance versus biological relevance. Statistical significance persisted in none of the observed associations after FDR correction, yet significant associations were confirmed in four of the seven genes selected for technical validation. These results suggest that the anti‐inflammatory intervention may affect the methylation status of metabolic genes in obese adolescents. However, further investigation is warranted to determine whether these alterations in DNA methylation mediate or are a consequence of phenotypic response.

The interrelationship between adiponectin and insulin sensitivity is interesting. Thiazolidinedione treatment is known to increase adiponectin in adults with T2D, but only 50–70% also show improved insulin sensitivity.^50^, ^51^ Similarly, we observed considerable heterogeneity in IR response to the AINS. Stratification of participants according to their magnitude of HOMA‐IR response revealed two distinct groups; responders and nonresponders. Despite equivalent compliance to the AINS, responders had significantly lower HOMA‐IR following the AINS, while nonresponders did not. Improved HOMA‐IR in responders was due to lower fasting insulin but not glucose concentrations; suggesting improved insulin signaling in metabolic tissues.

Within the personalized nutrition paradigm, the objective is to identify biomarkers of risk and response from the baseline metabolic phenotype. To this end, our group and others have demonstrated that baseline dietary, anthropometry, physical activity, biochemistry, and gut microbiota explain up to half of the variability in intervention response.^27^, ^52^ Remarkably although responders and nonresponders had comparable BMI, responder's baseline metabolic phenotype was indicative of an adverse metabolic phenotype. At baseline, responders had higher insulin, total and LDL cholesterol concentrations, than nonresponders with equivalent age, gender, BMI, and glucose concentrations. Interestingly, a similar pattern of response was demonstrated in children who underwent a lifestyle‐induced weight loss program.^52^ While responders had lower fasting glucose and TAG concentrations following intervention, 45% of children did not exhibit lower glucose or TAG levels despite similar weight loss.^52^ Both groups had comparable BMI and body composition; however, responders had higher fasting insulin, HOMA‐IR, fasting glucose, and 2 hour glucose pretreatment.^52^ Optimal cutpoints for four biomarkers could successfully predict responders from nonresponders with good specificity and sensitivity. While it would be prudent to verify these cutpoints in independent studies, these biomarker levels may be useful at predicting those who will successfully respond to anti‐inflammatory intervention and may aid participant selection for future trials. Adolescence may represent a unique window of metabolic plasticity during which heightened responsiveness is observed in high‐risk individuals.

While the strength of this study lies in its robust study design, some limitations exist. Firstly, study participants were predominantly insulin sensitive which may have limited potential efficacy of the AINS intervention. Also, HOMA‐IR is a useful fasting, but nevertheless limited, surrogate measure of IR. Postprandial glycemic control can vary greatly, which is not always apparent in the fasted state.^27^ Therefore, it is plausible that HOMA‐IR underestimated the efficacy of the anti‐inflammatory intervention. Further work should determine whether the AINS could also improve the postprandial response to an oral glucose or lipid tolerance test.

In conclusion, the results of this study are highly relevant within the current prevalence of pediatric obesity. Here we demonstrated that an AINS did not alter IR but did maintain HMW adiponectin and upregulate PBMC adiponectin receptor mRNA expression in overweight and obese adolescents. Furthermore, we showed that the HMW adiponectin response was related to epigenetic modulation of adipogenic genes. From the personalized perspective, the anti‐inflammatory nutrients attenuated obesity‐induced IR in those adolescents with an adverse baseline metabolic phenotype. Importantly, these improvements were observed in the absence of weight loss. Given the challenges associated with weight management, this may represent an attractive preventative measure, which focuses on nutrient inclusion rather than energy restriction.

## Conflict of Interest

The authors declare no conflict of interest.

## Supporting information

Supporting Materials
