# Supplementary material for: Personalized Cardio‐Metabolic Responses to an Anti‐Inflammatory Nutrition Intervention in Obese Adolescents: A Randomized Controlled Crossover Trial
Source: Mol Nutr Food Res. 2018 May 17;62(10):1701008. doi: 10.1002/mnfr.201701008 (PMC6079645; doi:10.1002/mnfr.201701008)
Supplement: Supplementary file 1 — Supporting Materials [file MNFR-62-na-s001.docx]

**Online**

**Supplemental Material**

| **Supplemental Table 1: Baseline characteristics of participants who completed (n=58) and discontinued (n=12) the trial** | | | | | |
| --- | --- | --- | --- | --- | --- |
|  | **Completed** |  | **Discontinued** |  | **P-value** |
| **N** | 58 |  | 12 |  |  |
| **Male:female (%)** | 33:67 |  | 40:60 |  | 0.682 |
| **Age (years)** | 15.9 (1.6) |  | 14.5 (1.6) |  | 0.007 |
| **Weight (kg)** | 93.02 (22.55) |  | 80.61 (14.06) |  | 0.068 |
| **Weight SDS** | 2.80 (1.09) |  | 2.58 (0.91) |  | 0.517 |
| **Height (cm)** | 169.73 (8.56) |  | 163.71 (7.38) |  | 0.027 |
| **Height SDS** | 0.64 (0.92) |  | 0.56 (0.90) |  | 0.793 |
| **BMI (kg/m^2^)** | 31.9 (4.4) |  | 30.9 (5.2) |  | 0.720 |
| **BMI SDS** | 2.6 (0.7) |  | 2.5 (0.7) |  | 0.684 |
| **Waist circumference (cm)** | 105.79 (14.07) |  | 100.00 (12.22) |  | 0.184 |
| **Waist circumference SDS** | 3.60 (0.88) |  | 3.69 (0.77) |  | 0.747 |
| **Waist:height ratio** | 0.62 (0.08) |  | 0.61 (0.08) |  | 0.705 |
| **Fat mass (kg)** | 35.97 (16.82) |  | 30.72 (11.54) |  | 0.960 |
| **Body fat %** | 37.62 (8.83) |  | 37.18 (8.23) |  | 0.874 |
| **Body fat % SDS** | 2.39 (0.84) |  | 2.28 (0.76) |  | 0.681 |
| **Fat mass index (kg/m^2^)** | 12.54 (5.66) |  | 11.58 (4.78) |  | 0.629 |
| **Fat free mass (kg)** | 56.15 (11.07) |  | 49.85 (5.68) |  | 0.118 |
| **Fat free mass index (kg/m^2^)** | 19.44 (2.41) |  | 18.56 (1.22) |  | 0.311 |
| **Muscle mass (kg)** | 53.14 (10.97) |  | 46.79 (5.17) |  | 0.141 |
| **Fat:fat free mass ratio** | 0.65 (0.29) |  | 0.62 (0.23) |  | 0.843 |
| a) Data are presented as mean (SD)  b) Data were analyzed by independent samples t-test between participants who completed and discontinued the trial  c) BMI (kg/m^2^) = weight (kg)/ height (m^2^); fat mass index (kg/m^2^) = fat mass (kg)/ height (m^2^); fat free mass index (kg/m^2^) = fat free mass (kg)/ height (m^2^),  d) SDS, age- and gender-normalized standard deviation score (UK 1990 Growth Reference Data) | | | | | |

| **Supplemental Table 2: Body composition and metabolic phenotype at baseline and follow-up in responders (n=23)** | | | | | | | | | |
| --- | --- | --- | --- | --- | --- | --- | --- | --- | --- |
|  | **AINS** | | |  | **Placebo** | | |  |  |
|  | **Baseline** |  | **Follow-up** |  | **Baseline** |  | **Follow-up** |  | **P-value** |
| **Body Composition** |  |  |  |  |  |  |  |  |  |
| **Weight (kg)** | 94·77 (24·55) |  | 94·51 (23·82) |  | 94·72 (25·26) |  | 94·69 (24·10) |  | 0·745 |
| **Body mass index (kg/m^2^)** | 33.0 (7·0) |  | 32·7 (6·8) |  | 33.0 (7·4) |  | 32·8 (7.0) |  | 0·576 |
| **Waist circumference (cm)** | 106·74 (13·98) |  | 105·91 (13·59) |  | 106·35 (15·99) |  | 106·30 (15·68) |  | 0·398 |
| **Fat mass (kg)** | 37·64 (15·36) |  | 36·87 (13·70) |  | 38·31 (17·25) |  | 37·80 (16·33) |  | 0·866 |
| **Body fat %** | 38·84 (6·88) |  | 38·48 (6·35) |  | 38·77 (6·61) |  | 38·61 (7·66) |  | 0·864 |
| **Fat mass index (kg/m^2^)** | 13·13 (5·09) |  | 12·80 (4·66) |  | 13·35 (5·79) |  | 13·10 (5·37) |  | 0·790 |
| **Fat free mass (kg)** | 57·12 (12·70) |  | 57·48 (13·10) |  | 56·38 (11·74) |  | 57·06 (11·22) |  | 0·616 |
| **Fat free mass index (kg/m^2^)** | 19·83 (2·93) |  | 19·80 (3·03) |  | 19·62 (2·65) |  | 19·78 (2·55) |  | 0·424 |
| **Fat:fat free mass ratio** | 0·66 (0·21) |  | 0·64 (0·18) |  | 0·67 (0·25) |  | 0·65 (0·22) |  | 0·718 |
| **Metabolic Phenotype** |  |  |  |  |  |  |  |  |  |
| **Glucose (mmol/L)** | 5·22 (0·36) |  | 5·07 (0·41) |  | 5·20 (0·44) |  | 5·12 (0·41) |  | 0·355 |
| **Insulin (mU/L)** | 14·86 (8·43) |  | 9·74 (4·56) |  | 12·83 (9·31) |  | 11·99 (6·26) |  | 0·003 |
| **HOMA-IR** | 3·49 (2·10) |  | 2·24 (1·18) |  | 3·04 (2·42) |  | 2·78 (1·63) |  | 0·004 |
| **HOMA-β (%)** | 172·82 (87·67) |  | 126·45 (52·03) |  | 149·47 (84·38) |  | 149·10 (62·01) |  | 0·015 |
| **QUICKI** | 0·32 (0·02) |  | 0·35 (0·02) |  | 0·34 (0·03) |  | 0·33 (0·02) |  | 0·001 |
| **TAG (mmol/L)** | 1·01 (0·49) |  | 0·96 (0·54) |  | 1·06 (0·48) |  | 1·21 (0·55) |  | 0·064 |
| **Fetuin A (μg/ml)** | 504·78 (309·34) |  | 670·38 (470·44) |  | 524·51 (323·91) |  | 629·17 (355·43) |  | 0.320 |
| **NEFA (mEq/L)** | 0·47 (0·17) |  | 0·64 (0·35) |  | 0·60 (0·29) |  | 0·56 (0·23) |  | 0·824 |
| **Total Cholesterol (mmol/L)** | 4·05 (0·74) |  | 4·09 (0·66) |  | 4·17 (0·74) |  | 3·99 (0·67) |  | 0·257 |
| **HDL Cholesterol (mmol/L)** | 1·25 (0·22) |  | 1·27 (0·25) |  | 1·27 (0·31) |  | 1·21 (0·17) |  | 0·328 |
| **LDL Cholesterol (mmol/L)** | 2·34 (0·56) |  | 2·39 (0·46) |  | 2·42 (0·57) |  | 2·23 (0·50) |  | 0·295 |
| **LDL:HDL ratio** | 1·92 (0·54) |  | 1·97 (0·56) |  | 2·09 (1·10) |  | 1·87 (0·47) |  | 0·525 |
| **APO A1 (mg/dL)** | 114·11(16·12) |  | 110·87 (20·66) |  | 117·48 (20·21) |  | 113·80 (16·11) |  | 0·903 |
| **Total Adiponectin (μg/ml)** | 7·4 (4·8) |  | 7·9 (5·5) |  | 7·1 (3·9) |  | 7·1 (3·9) |  | 0·733 |
| **HMW Adiponectin (μg/ml)** | 3·7 (2·8) |  | 4·2 (3·4) |  | 3·8 (2·3) |  | 3·3 (2·5) |  | 0·056 |
| **Leptin (ng/ml)** | 37·6 (24·0) |  | 32·3 (21·1) |  | 40·7 (29·5) |  | 37·7 (20·7) |  | 0·616 |
| **FABP4 (ng/ml)** | 28·1 (26·3) |  | 27·1 (14·9) |  | 23·2 (14·1) |  | 27·9 (14·2) |  | 0·283 |
| **sCD163 (ng/ml)** | 702.0(293·4) |  | 689·8 (272.0) |  | 675·7 (311·4) |  | 650·3 (251·7) |  | 0·796 |
| **Complement C3 (mg/ml)** | 0·7 (0·2) |  | 0·5 (0·3) |  | 0·6 (0·4) |  | 0·6 (0·4) |  | 0·318 |
| a) Data are presented as mean (SD),  b) Data were analyzed using paired samples t-tests between delta supplement and delta placebo values  c) ApoA1, apolipoprotein A1; FABP4, fatty acid binding protein 4; HDL; HOMA-IR, homeostatic model assessment- insulin resistance; HOMA-β, homeostatic model assessment- β-cell function; LDL; NEFA, non-esterified fatty acids; QUICKI, quantitative insulin sensitivity check index; TAG, triacylglycerol. | | | | | | | | | |

| **Supplemental Table 3: Cholesteryl ester fatty acid composition and plasma antioxidant status at baseline and follow-up (n=58)** | | | | | | | | | | |
| --- | --- | --- | --- | --- | --- | --- | --- | --- | --- | --- |
|  |  | **AINS** | | |  | **Placebo** | | |  |  |
|  |  | **Baseline** |  | **Follow-up** |  | **Baseline** |  | **Follow-up** |  | **P-value** |
| **Fatty acid profile (% total fatty acids)** | | | | | | | | | | |
| **SFA** |  | 30·32 (10·67) |  | 30·76 (11·25) |  | 31·92 (11·62) |  | 31·66 (10·08) |  | 0·793 |
| **C16:0** |  | 26·48 (13·22) |  | 29·24 (11·14) |  | 30·40 (11·84) |  | 30·25 (10·17) |  | 0·170 |
| **C18:0** |  | 1·34 (0·56) |  | 1·40 (0·65) |  | 1·52 (1·10) |  | 1·41 (0·62) |  | 0·315 |
| **MUFA** |  | 17·29 (5·30) |  | 16·76 (5·26) |  | 16·49 (5·03) |  | 16·96 (5·35) |  | 0·282 |
| **C16:1n-7** |  | 6·01 (1·65) |  | 6·09 (1·80) |  | 5·87 (2·02) |  | 5·80 (1·88) |  | 0·590 |
| **C18:1n-9** |  | 11·28 (3·83) |  | 10·66 (3·64) |  | 10·62 (3·41) |  | 11·16 (3·74) |  | 0·063 |
| **PUFA** |  | 42·42 (9·37) |  | 41·86 (8·78) |  | 40·98 (8·92) |  | 41·33 (8·86) |  | 0·985 |
| **C18:2n-6** |  | 33·29 (9·18) |  | 31·73 (8·49) |  | 31·93 (9·15) |  | 31·88 (8·68) |  | 0·596 |
| **C20:3n-6** |  | 1·01 (0·67) |  | 1·09 (0·89) |  | 1·17 (0·76) |  | 1·15 (0·78) |  | 0·529 |
| **C20:4n-6** |  | 4·39 (1·63) |  | 4·07 (1·80) |  | 3·98 (1·33) |  | 4·24 (1·69) |  | 0·046 |
| **C18:3n-3** |  | 1·32 (0·84) |  | 1·36 (0·87) |  | 1·28 (0·81) |  | 1·40 (0·87) |  | 0·957 |
| **C20:5n-3** |  | 0·81 (0·56) |  | 1·50 (0·77) |  | 0·89 (0·47) |  | 0·89 (0·51) |  | <0·001 |
| **C22:6n-3** |  | 1·60 (1·17) |  | 2·19 (1·29) |  | 1·74 (1·25) |  | 1·77 (1·29) |  | 0·042 |
| **Total n-6** |  | 38·70 (9·91) |  | 36·79 (9·12) |  | 37·07 (9·73) |  | 37·27 (9·44) |  | 0·451 |
| **Total n-3** |  | 3·72 (1·69) |  | 5·07 (1·79) |  | 3·91 (1·87) |  | 4·06 (2·01) |  | 0.002 |
| **PUFA:SFA** |  | 1·69 (0·91) |  | 1·63 (0·82) |  | 1·55 (0·81) |  | 1·56 (0·89) |  | 0·858 |
| **Anti-oxidant status (μmol/L)** | | | |  |  |  |  |  |  |  |
| **α-tocopherol** |  | 18·26 (6·80) |  | 21·19 (8·11) |  | 19·86 (8·13) |  | 19·76 (8·09) |  | 0·045 |
| **Lycopene** |  | 0·70 (0·52) |  | 1·00 (0·67) |  | 0·72 (0·52) |  | 0·81 (0·60) |  | 0·014 |
| a) Data are presented as mean (SD)  b) Data were analyzed using paired samples t-tests between delta supplement and delta placebo values  c) MUFA, monounsaturated fatty acids; PUFA, polyunsaturated fatty acids; SFA, saturated fatty acids | | | | | | | | | | |

| Supplemental Table 4 Estimates of the predictive accuracy of significant biomarkers differentiating responders from non-responders to the AINS intervention. | | | | | | |
| --- | --- | --- | --- | --- | --- | --- |
|  | **AUC (95% C.I.)** | **P value** | **Optimal cutpoint** | **LR** | **Sensitivity** | **Specificity** |
| **HOMA-IR** | 0.73 (0.60, 0.86) | 0.0029 | 2.39 | 1.68 | 65.7 | 60.9 |
| **HOMA-B** | 0.75 (0.63, 0.88) | 0.001 | 111.2% | 4.4 | 60.0 | 86.3 |
| **Insulin** | 0.75 (0.63, 0.87) | 0.0009 | 9.57mU/L | 2.52 | 65.7 | 73.9 |
| **QUICKI** | 0.73 (0.61, 0.86) | 0.002 | 0.34 | 1.75 | 68.6 | 60.9 |
| **Total Cholesterol** | 0.66 (0.51, 0.80) | 0.047 | 3.83mmol/L | 1.75 | 68.6 | 60.9 |
| **LDL Cholesterol** | 0.66 (0.52, 0.81) | 0.036 | 2.05mmol/L | 1.52 | 69.6 | 54.3 |
| AUC values (95% confidence intervals) were derived for individual biomarker values, the highest likelihood ratio test indicated the optimal cutpoint for each biomarker along with sensitivity and specificity. HOMA-IR, homeostatic model assessment- insulin resistance; HOMA-β, homeostatic model assessment- β-cell function; LDL; QUICKI, quantitative insulin sensitivity check index; LDL, low density lipoprotein; AUC, area under the curve; LR, likelihood ratio. | | | | | | |

| **Supplemental Table 5: DNA methylation changes as they relate to HMW adiponectin response following the anti-inflammatory nutrition supplement as assessed by Illumina (n=55)** | | | | | | | |
| --- | --- | --- | --- | --- | --- | --- | --- |
| **Gene Name** | **Chromosome Position** | **Correlation Co-efficient** | **P-Value** | **Gene Name** | **Chromosome Position** | **Correlation Co-efficient** | **P-Value** |
| **Positive Associations** | | |  |  |  |  |  |
| TNIP3 | chr4:122138632 | 0.5486752 | 3.064E-05 | C1orf93 | chr1:2520499 | 0.41915211 | 0.0022026 |
| STYXL1 | chr7:75677469 | 0.5397792 | 4.356E-05 | CHST6 | chr16:75529892 | 0.41887292 | 0.0022192 |
|  | chrY:15866837 | 0.5140961 | 0.0001139 | CNNM2 | chr10:104678015 | 0.41847337 | 0.0022431 |
| RNASEH1 | chr2:3606104 | 0.5062339 | 0.0001506 | CUGBP2 | chr10:11207907 | 0.41843521 | 0.0022454 |
| C5orf54 | chr5:159826550 | 0.4922418 | 0.0002435 | COMMD5 | chr8:146077906 | 0.41835661 | 0.0022502 |
| RNF13 | chr3:149567827 | 0.4887257 | 0.0002739 | CCR3 | chr3:46307918 | 0.41753546 | 0.0023003 |
| ATP8B3 | chr19:1792217 | 0.4868053 | 0.0002919 | GINS2 | chr16:85723489 | 0.41650531 | 0.0023645 |
| CYB5R3 | chr22:43037594 | 0.4828394 | 0.0003325 | KIAA0182 | chr16:85670348 | 0.41637595 | 0.0023727 |
| STARD10 | chr11:72479131 | 0.4771697 | 0.0003995 | USP43 | chr17:9549346 | 0.41533977 | 0.0024391 |
|  | chr3:196048411 | 0.4665494 | 0.0005587 |  | chr5:92938232 | 0.41531168 | 0.002441 |
|  | chr4:78905783 | 0.4642156 | 0.0006006 | C1orf159 | chr1:1024413 | 0.41521377 | 0.0024473 |
| MGMT | chr10:131264264 | 0.4640859 | 0.000603 | UBAC2 | chr13:99853132 | 0.41431264 | 0.0025066 |
|  | chr1:228741287 | 0.4604717 | 0.0006737 | INPP5D | chr2:233925923 | 0.41421703 | 0.002513 |
| IRX2 | chr5:2751822 | 0.459015 | 0.0007042 | TTTY18 | chrY:8551551 | 0.41417943 | 0.0025155 |
| KIAA1549 | chr7:138556982 | 0.4563168 | 0.0007641 | ZNF709 | chr19:12595882 | 0.41326591 | 0.0025771 |
|  | chr1:228891036 | 0.4554919 | 0.0007833 |  | chr22:29464693 | 0.4119119 | 0.0026708 |
| DYSF | chr2:71693579 | 0.4530687 | 0.0008422 | WNT1 | chr12:49375669 | 0.4117818 | 0.00268 |
| LOC100133161 | chr11:129632 | 0.4526028 | 0.000854 | ZNF677 | chr19:53754612 | 0.4104297 | 0.0027769 |
|  | chr14:101924596 | 0.4494084 | 0.0009388 | MED13L | chr12:116581414 | 0.4101899 | 0.0027944 |
| CD93 | chr20:23066944 | 0.4475642 | 0.0009911 |  | chr4:1604486 | 0.4094379 | 0.00285 |
| SNORD113-9 | chr14:101411642 | 0.4453375 | 0.0010577 | PRDX6 | chr1:173446315 | 0.4094359 | 0.0028501 |
|  | chr15:94346356 | 0.4429223 | 0.0011346 | CACNB1 | chr17:37354028 | 0.4084547 | 0.002924 |
| MYOM2 | chr8:2020566 | 0.4423653 | 0.001153 | RCOR3 | chr1:211434045 | 0.4083916 | 0.0029289 |
| ARHGEF10 | chr8:1831484 | 0.4401952 | 0.0012273 |  | chr12:65350145 | 0.4083091 | 0.0029352 |
| PYGL | chr14:51406877 | 0.4391699 | 0.0012638 | FMR1NB | chrX:147062704 | 0.4080363 | 0.0029561 |
| PDSS2 | chr6:107780783 | 0.4383567 | 0.0012935 | CDK5R1 | chr17:30813443 | 0.4077125 | 0.0029811 |
|  | chr17:6950103 | 0.4380317 | 0.0013056 | C2orf14 | chr2:131443311 | 0.4075166 | 0.0029963 |
| SLFN12 | chr17:33759484 | 0.4378009 | 0.0013142 | C6orf122 | chr6:170190660 | 0.4069913 | 0.0030375 |
| SGPP2 | chr2:223419421 | 0.4375744 | 0.0013227 |  | chr14:102418598 | 0.4063929 | 0.003085 |
| MGLL | chr3:127543493 | 0.4365107 | 0.0013633 | MED4 | chr13:48669363 | 0.4057923 | 0.0031333 |
| C2orf62 | chr2:219221131 | 0.436414 | 0.001367 | NAA15 | chr4:140223063 | 0.4053908 | 0.0031659 |
| ORAOV1 | chr11:69490215 | 0.4345636 | 0.0014405 |  | chr17:43451392 | 0.4053433 | 0.0031698 |
| MKNK2 | chr19:2051251 | 0.4327124 | 0.0015174 |  | chr20:48624433 | 0.4042215 | 0.0032628 |
| MRPL3 | chr3:131218171 | 0.4326074 | 0.0015219 | GSTT1 | chr22:24384159 | 0.4024198 | 0.0034172 |
| BAI1 | chr8:143616587 | 0.4319343 | 0.0015509 | PLEKHG5 | chr1:6551073 | 0.4020427 | 0.0034503 |
|  | chr6:68599280 | 0.4288474 | 0.0016901 | EYA1 | chr8:72130951 | 0.4019871 | 0.0034552 |
| RAB21 | chr12:72148853 | 0.4275165 | 0.0017535 | ANKS1A | chr6:34856217 | 0.4018661 | 0.003466 |
|  | chr7:56877538 | 0.4263129 | 0.0018126 | PLAC8 | chr4:84031278 | 0.4017610 | 0.0034753 |
| RPTOR | chr17:78803474 | 0.425375 | 0.0018599 | C7orf13 | chr7:156433520 | 0.4016489 | 0.0034852 |
| MTA1 | chr14:105936409 | 0.42535 | 0.0018612 | C5orf38 | chr5:2753876 | 0.4012873 | 0.0035175 |
| ZNF398 | chr7:148848205 | 0.4223952 | 0.0020176 |  | chr16:22960289 | 0.4011699 | 0.0035281 |
| FAM194B | chr13:46159050 | 0.4218159 | 0.0020496 | ALDH3B2 | chr11:67432882 | 0.4010720 | 0.0035369 |
|  | chr3:195578040 | 0.4199809 | 0.0021539 |  |  |  |  |

| **Supplemental Table 5 continued: DNA methylation changes as they relate to HMW adiponectin response following the anti-inflammatory nutrition supplement as assessed by Illumina (n=55)** | | | | | | | |
| --- | --- | --- | --- | --- | --- | --- | --- |
| **Gene Name** | **Chromosome Position** | **Correlation Co-efficient** | **P-Value** | **Gene Name** | **Chromosome Position** | **Correlation Co-efficient** | **P-Value** |
| **Inverse Associations** | | |  |  |  |  |  |
| GPC6 | chr13:95003984 | -0.5800018 | 8.173E-06 | PSMB11 | chr14:23512002 | -0.4674141 | 0.0005439 |
| CHRNA6 | chr8:42623730 | -0.5468168 | 3.301E-05 | FGF6 | chr12:4554463 | -0.4673166 | 0.0005455 |
| SORCS2 | chr4:7666180 | -0.5393561 | 4.428E-05 | BLCAP | chr20:36154222 | -0.467252 | 0.0005466 |
| FAM114A1 | chr4:38872591 | -0.5333788 | 5.576E-05 | SERPING1 | chr11:57363540 | -0.467028 | 0.0005505 |
| VWA1 | chr1:1370775 | -0.5311411 | 6.071E-05 |  | chr4:1041664 | -0.4661862 | 0.000565 |
| POLR1A | chr2:86282139 | -0.5242922 | 7.85E-05 |  | chr5:12230982 | -0.4659524 | 0.0005692 |
|  | chr4:3899366 | -0.5194931 | 9.368E-05 | SLC39A3 | chr19:2732657 | -0.4655698 | 0.0005759 |
| KLF14 | chr7:130419340 | -0.5133992 | 0.0001168 | PPFIA2 | chr12:81763492 | -0.4654881 | 0.0005774 |
| GRN | chr17:42430376 | -0.5124667 | 0.0001208 | CACNA1C | chr12:2800755 | -0.463972 | 0.0006051 |
| NPR1 | chr1:153652247 | -0.511321 | 0.0001258 | STK32B | chr4:5129810 | -0.4624816 | 0.0006335 |
| DLGAP2 | chr8:1462183 | -0.5102019 | 0.0001309 | DDX12 | chr12:9600888 | -0.4595513 | 0.0006928 |
|  | chr8:127479891 | -0.5085121 | 0.000139 | PRKY | chrY:7140718 | -0.457876 | 0.0007289 |
|  | chr2:168151004 | -0.5035 | 0.0001657 |  | chr1:61145559 | -0.4578365 | 0.0007298 |
| GAB2 | chr11:78052863 | -0.5029103 | 0.0001692 |  | chr5:3539154 | -0.4576461 | 0.000734 |
| HCCA2 | chr11:1647150 | -0.4977053 | 0.0002024 | CYP2D7P1 | chr22:42537241 | -0.457389 | 0.0007397 |
| HLA-A | chr6:29911036 | -0.4973405 | 0.0002049 | GCN1L1 | chr12:120631901 | -0.4571804 | 0.0007444 |
| KHDRBS2 | chr6:62737607 | -0.495302 | 0.0002196 | CD2 | chr1:117296494 | -0.4561282 | 0.0007684 |
|  | chr6:148020881 | -0.4945363 | 0.0002254 | HDAC4 | chr2:240274542 | -0.4559223 | 0.0007732 |
|  | chr8:94133100 | -0.4942867 | 0.0002273 | SETD1B | chr12:122250153 | -0.4558313 | 0.0007753 |
|  | chr11:125951005 | -0.4905988 | 0.0002573 |  | chr4:16362584 | -0.4552627 | 0.0007887 |
| C20orf24 | chr20:35238351 | -0.4903193 | 0.0002597 | CNNM2 | chr10:104724237 | -0.4551351 | 0.0007917 |
| MACF1 | chr1:39873077 | -0.4894457 | 0.0002674 |  | chr11:23997506 | -0.4549294 | 0.0007966 |
| HIC1 | chr17:1958164 | -0.4871623 | 0.0002885 | PLA2G4E | chr15:42302705 | -0.4535951 | 0.0008291 |
| TTC28 | chr22:28402098 | -0.4866004 | 0.0002939 | MIR150 | chr19:50005045 | -0.4531945 | 0.000839 |
|  | chr15:95287886 | -0.4835484 | 0.0003249 | C13orf15 | chr13:42031584 | -0.4519830 | 0.0008699 |
| CCNDBP1 | chr15:43477701 | -0.4829928 | 0.0003309 | UTS2 | chr1:7913865 | -0.4519355 | 0.0008711 |
|  | chr14:103655424 | -0.4820603 | 0.0003411 | PLEKHM1P | chr17:62833709 | -0.4513011 | 0.0008876 |
| GTF2H4 | chr6:30875752 | -0.4819197 | 0.0003427 | FAT1 | chr4:187541541 | -0.4509475 | 0.000897 |
| MICB | chr6:31474171 | -0.4793868 | 0.000372 |  | chr16:85336004 | -0.4496632 | 0.0009317 |
|  | chr10:118591023 | -0.478758 | 0.0003796 | C6orf41 | chr6:26923983 | -0.4495680 | 0.0009344 |
|  | chr1:170278698 | -0.4786133 | 0.0003814 | ST14 | chr11:130076290 | -0.4494681 | 0.0009371 |
| DGKG | chr3:186080015 | -0.4782902 | 0.0003854 | PIAS1 | chr15:68370885 | -0.4490546 | 0.0009486 |
| NAPA | chr19:48018641 | -0.4778416 | 0.000391 | MCM10 | chr10:13203437 | -0.4488288 | 0.0009549 |
| SYNJ1 | chr21:34100309 | -0.4773325 | 0.0003974 |  | chr8:82958662 | -0.4486073 | 0.0009612 |
|  | chr3:128572244 | -0.4766794 | 0.0004059 | EEF1DP3 | chr13:32526883 | -0.4478921 | 0.0009816 |
| COL4A2 | chr13:110988307 | -0.4766026 | 0.0004069 | HCP5 | chr6:31430641 | -0.4469739 | 0.0010084 |
| SLIT3 | chr5:168245286 | -0.473841 | 0.0004443 | FAM118B | chr11:126114136 | -0.4469703 | 0.0010085 |
|  | chr17:76250004 | -0.4736714 | 0.0004467 | GNAT1 | chr3:50228220 | -0.4465852 | 0.0010199 |
| ACSL3 | chr2:223725459 | -0.4707265 | 0.0004903 | TCF23 | chr2:27371642 | -0.4465234 | 0.0010218 |
| RP1 | chr8:55533939 | -0.4701104 | 0.0004999 |  | chr15:31420951 | -0.4464820 | 0.001023 |
| SOX5 | chr12:23714914 | -0.4698485 | 0.000504 | HPS1 | chr10:100206925 | -0.4460692 | 0.0010354 |
| MLF1IP | chr4:185655609 | -0.4686084 | 0.000524 | PDCD5 | chr19:33072785 | -0.4459467 | 0.0010391 |
|  | chr4:42331783 | -0.4681735 | 0.0005311 | CTNNA2 | chr2:80179759 | -0.444544 | 0.0010825 |

| **Supplemental Table 5 continued: DNA methylation changes as they relate to HMW adiponectin response following the anti-inflammatory nutrition supplement as assessed by Illumina (n=55)** | | | | | | | |
| --- | --- | --- | --- | --- | --- | --- | --- |
| **Gene Name** | **Chromosome Position** | **Correlation Co-efficient** | **P-Value** | **Gene Name** | **Chromosome Position** | **Correlation Co-efficient** | **P-Value** |
| **Inverse Associations** | | |  |  |  |  |  |
| MYO5C | chr15:52546043 | -0.4445377 | 0.0010827 | CALD1 | chr7:134463438 | -0.4333184 | 0.0014918 |
| GFAP | chr17:42994269 | -0.4442157 | 0.0010928 |  | chr17:21937235 | -0.4331943 | 0.0014971 |
| TAPT1 | chr4:16214868 | -0.4439995 | 0.0010997 |  | chr2:392873 | -0.4328906 | 0.0015099 |
| LSM2 | chr6:31766239 | -0.4431337 | 0.0011276 | LOC100131193 | chr9:139702200 | -0.4328782 | 0.0015104 |
| C2orf70 | chr2:26785460 | -0.4425597 | 0.0011465 | ZDHHC1 | chr16:67430454 | -0.4328641 | 0.001511 |
| NAT10 | chr11:34127610 | -0.4421903 | 0.0011588 |  | chr8:79027162 | -0.4323475 | 0.001533 |
| FBRSL1 | chr12:133118770 | -0.4419722 | 0.0011661 |  | chr1:116467163 | -0.4320579 | 0.0015455 |
| PPBPL2 | chr4:74921023 | -0.4418603 | 0.0011699 | CDC42EP3 | chr2:37882358 | -0.431347 | 0.0015765 |
|  | chr19:13266516 | -0.4418588 | 0.0011699 | PLXNA2 | chr1:208382186 | -0.4310816 | 0.0015883 |
|  | chr19:36795950 | -0.441245 | 0.0011908 | LAMA2 | chr6:129677367 | -0.4309083 | 0.001596 |
|  | chrX:113813553 | -0.4412182 | 0.0011917 | BCLAF1 | chr6:136607649 | -0.4307896 | 0.0016013 |
| MIR519D | chr19:54215458 | -0.441147 | 0.0011942 | ADARB2 | chr10:1247469 | -0.4301775 | 0.0016288 |
|  | chr15:89952052 | -0.4410094 | 0.0011989 | MDC1 | chr6:30685074 | -0.4297465 | 0.0016484 |
|  | chr11:102430004 | -0.4409679 | 0.0012003 | C16orf88 | chr16:19718086 | -0.4296617 | 0.0016523 |
| CACNA2D3 | chr3:54737888 | -0.4409432 | 0.0012012 | RASSF6 | chr4:74486179 | -0.4295298 | 0.0016584 |
| CEMP1 | chr16:2582813 | -0.4408767 | 0.0012035 |  | chr6:31055396 | -0.4291854 | 0.0016743 |
|  | chr4:185871509 | -0.4407985 | 0.0012062 | DYNC1I1 | chr7:95546508 | -0.4289417 | 0.0016857 |
|  | chr5:56820679 | -0.4406567 | 0.0012111 | C1orf63 | chr1:25574015 | -0.4289130 | 0.001687 |
| PANX2 | chr22:50608598 | -0.4404519 | 0.0012183 | SLC43A1 | chr11:57283212 | -0.4288130 | 0.0016917 |
| MALT1 | chr18:56337165 | -0.4397792 | 0.001242 | GABRB3 | chr15:26896681 | -0.4287485 | 0.0016947 |
| DIP2C | chr10:532908 | -0.4397489 | 0.0012431 |  | chr13:44868172 | -0.4287465 | 0.0016948 |
|  | chr4:3647047 | -0.4394206 | 0.0012548 | CHDH | chr3:53855929 | -0.4281805 | 0.0017216 |
| ABP1 | chr7:150554420 | -0.439325 | 0.0012583 | YKT6 | chr7:44240498 | -0.4280691 | 0.0017269 |
|  | chr8:75087963 | -0.439242 | 0.0012612 |  | chr5:140577279 | -0.4278654 | 0.0017367 |
| JAKMIP3 | chr10:133960405 | -0.4391407 | 0.0012649 | CREB1 | chr2:208393829 | -0.4277274 | 0.0017433 |
| DSG2 | chr18:29080265 | -0.4388951 | 0.0012738 |  | chr8:67873476 | -0.4277271 | 0.0017433 |
| VSTM2B | chr19:30018436 | -0.4381853 | 0.0012999 | PYHIN1 | chr1:158900549 | -0.4277071 | 0.0017443 |
|  | chr9:137387895 | -0.4377507 | 0.0013161 | ALOX5 | chr10:45868696 | -0.4276786 | 0.0017456 |
| DACH2 | chrX:85407140 | -0.4376919 | 0.0013183 |  | chr1:5771047 | -0.4276701 | 0.001746 |
| FAM135A | chr6:71122946 | -0.4376059 | 0.0013215 |  | chr12:132956918 | -0.4275073 | 0.0017539 |
| PDZK1 | chr1:145726979 | -0.4375323 | 0.0013243 |  | chr9:116143143 | -0.4274584 | 0.0017563 |
|  | chr7:140023476 | -0.4374514 | 0.0013273 |  | chr4:185942701 | -0.4273787 | 0.0017602 |
|  | chr4:40193114 | -0.4373949 | 0.0013295 | ARHGAP20 | chr11:110578533 | -0.4273757 | 0.0017603 |
| DPP6 | chr7:154455543 | -0.4372033 | 0.0013367 | KIAA1486 | chr2:226265799 | -0.4273232 | 0.0017629 |
| ABR | chr17:952903 | -0.4362303 | 0.0013742 | SCAF1 | chr19:50151457 | -0.4270909 | 0.0017742 |
| ZNF704 | chr8:81787141 | -0.4356837 | 0.0013956 | SDK1 | chr7:4308209 | -0.4269401 | 0.0017816 |
|  | chr11:130710076 | -0.4356578 | 0.0013966 | TTC17 | chr11:43380526 | -0.4268046 | 0.0017882 |
| CACNA2D3 | chr3:54934591 | -0.4356378 | 0.0013974 | COL9A1 | chr6:71014153 | -0.4267806 | 0.0017894 |
| OR2AG1 | chr11:6804983 | -0.4355963 | 0.0013991 | SDF4 | chr1:1153338 | -0.4266782 | 0.0017945 |
| THBS2 | chr6:169650832 | -0.4346315 | 0.0014377 | LPCAT3 | chr12:7126256 | -0.4264990 | 0.0018033 |
| C18orf16 | chr18:24446444 | -0.4344163 | 0.0014465 |  | chr12:65939993 | -0.4264802 | 0.0018043 |
|  | chr20:2648594 | -0.4342183 | 0.0014546 | RBM12B | chr8:94753498 | -0.4260951 | 0.0018235 |
| PRKG1 | chr10:52754927 | -0.4336468 | 0.0014781 | FAM65B | chr6:24874614 | -0.426051 | 0.0018257 |

| **Supplemental Table 5 continued: DNA methylation changes as they relate to HMW adiponectin response following the anti-inflammatory nutrition supplement as assessed by Illumina (n=55)** | | | | | | | |
| --- | --- | --- | --- | --- | --- | --- | --- |
| **Gene Name** | **Chromosome Position** | **Correlation Co-efficient** | **P-Value** | **Gene Name** | **Chromosome Position** | **Correlation Co-efficient** | **P-Value** |
| **Inverse Associations** | | |  |  |  |  |  |
| SNAP47 | chr1:227953546 | -0.4259667 | 0.0018299 | SLIT1 | chr10:98799272 | -0.4195388 | 0.0021797 |
|  | chr14:74895911 | -0.4258346 | 0.0018366 | OSBPL10 | chr3:31977309 | -0.4194758 | 0.0021834 |
| ZBTB12 | chr6:31870259 | -0.4257461 | 0.0018411 |  | chr6:169244459 | -0.4193075 | 0.0021934 |
|  | chr16:4170088 | -0.4253738 | 0.00186 | OR6K3 | chr1:158688804 | -0.4192967 | 0.002194 |
| DUSP4 | chr8:29208240 | -0.4252343 | 0.0018671 | ZNF167 | chr3:44596384 | -0.4191778 | 0.002201 |
| KIF25 | chr6:168418527 | -0.4251296 | 0.0018725 | HMX3 | chr10:124895448 | -0.4191735 | 0.0022013 |
|  | chr1:90284686 | -0.4249905 | 0.0018796 |  | chr3:14615941 | -0.4189244 | 0.0022161 |
| RPL9 | chr4:39460522 | -0.4248125 | 0.0018888 |  | chr14:106113192 | -0.418712 | 0.0022288 |
|  | chr13:42136511 | -0.4248095 | 0.001889 | PRNP | chr20:4667405 | -0.4184821 | 0.0022426 |
| MCF2L | chr13:113672808 | -0.4246283 | 0.0018983 | D2HGDH | chr2:242695603 | -0.4182992 | 0.0022536 |
| STK32C | chr10:134096650 | -0.4245791 | 0.0019009 |  | chr5:42992555 | -0.4182035 | 0.0022594 |
| IRF8 | chr16:85932214 | -0.4244588 | 0.0019072 |  | chr11:49017551 | -0.4181775 | 0.002261 |
| OR8H2 | chr11:55872467 | -0.42442 | 0.0019092 | SEC23A | chr14:39572489 | -0.4179727 | 0.0022735 |
| C3orf37 | chr3:128997662 | -0.4242248 | 0.0019194 | GRM8 | chr7:126364460 | -0.4176205 | 0.002295 |
|  | chr5:170744407 | -0.4242029 | 0.0019206 | THEM4 | chr1:151878061 | -0.4176147 | 0.0022954 |
| TUBGCP2 | chr10:135121331 | -0.4236884 | 0.0019477 | MYO16 | chr13:109318335 | -0.4175900 | 0.0022969 |
| COL11A2 | chr6:33146085 | -0.4236305 | 0.0019508 |  | chr6:14316838 | -0.4172401 | 0.0023185 |
| TMCO7 | chr16:68942231 | -0.4235686 | 0.0019541 | BAIAP2L1 | chr7:97978655 | -0.4166898 | 0.0023529 |
| CDC42BPG | chr11:64594467 | -0.4235261 | 0.0019564 | C12orf40 | chr12:40019768 | -0.4163754 | 0.0023727 |
| MYF6 | chr12:81102123 | -0.4235256 | 0.0019564 |  | chr20:43372556 | -0.4163366 | 0.0023752 |
| MAFK | chr7:1579643 | -0.4231874 | 0.0019745 | PARK2 | chr6:162831837 | -0.4161779 | 0.0023853 |
|  | chr7:45035665 | -0.4229829 | 0.0019856 |  | chr4:41878739 | -0.4161641 | 0.0023862 |
|  | chr6:5030141 | -0.4229437 | 0.0019877 | C6orf122 | chr6:170198971 | -0.4159605 | 0.0023992 |
| PTRH1 | chr9:130477698 | -0.4229153 | 0.0019892 | TDRKH | chr1:151762605 | -0.4156858 | 0.0024168 |
| ZZEF1 | chr17:3960466 | -0.4228458 | 0.001993 | METTL10 | chr10:126481025 | -0.4156810 | 0.0024171 |
| PLRG1 | chr4:155470815 | -0.4228145 | 0.0019947 | AGBL1 | chr15:87155056 | -0.4154235 | 0.0024337 |
|  | chr4:186921049 | -0.4223323 | 0.002021 |  | chr8:1301014 | -0.4152807 | 0.002443 |
| SEMA3F | chr3:50196278 | -0.4218947 | 0.0020452 | UBE3A | chr15:25683909 | -0.4151866 | 0.0024491 |
|  | chr5:177484324 | -0.4218619 | 0.002047 |  | chr16:47999378 | -0.4151385 | 0.0024522 |
| ZFHX3 | chr16:72991344 | -0.4218064 | 0.0020501 | GAP43 | chr3:115373404 | -0.4150291 | 0.0024594 |
| AFF3 | chr2:100210037 | -0.4217133 | 0.0020553 | KIF7 | chr15:90200115 | -0.4149941 | 0.0024617 |
| ICT1 | chr17:73008733 | -0.4214481 | 0.0020701 | LOC550112 | chr4:68586568 | -0.4149741 | 0.002463 |
| GTF2A1L | chr2:48946375 | -0.4212107 | 0.0020835 | KCNK3 | chr2:26927436 | -0.4149092 | 0.0024672 |
|  | chr5:173846716 | -0.4211193 | 0.0020886 |  | chr20:17546270 | -0.4147049 | 0.0024807 |
| C16orf35 | chr16:189100 | -0.4209439 | 0.0020986 | CCDC54 | chr3:107097413 | -0.4146103 | 0.0024869 |
| C3orf21 | chr3:194807038 | -0.4208977 | 0.0021012 |  | chr2:88510846 | -0.4145051 | 0.0024939 |
| INSR | chr19:7293776 | -0.4208313 | 0.002105 | NT5C2 | chr10:104953200 | -0.4144870 | 0.0024951 |
| ZFYVE16 | chr5:79703734 | -0.4207406 | 0.0021101 | LOC100130093 | chr1:227915962 | -0.4143795 | 0.0025022 |
| PLCE1 | chr10:95796595 | -0.4204139 | 0.0021289 | NDUFA13 | chr19:19637361 | -0.4143392 | 0.0025049 |
| C20orf20 | chr20:61427998 | -0.4203739 | 0.0021312 | UGT1A10 | chr2:234652601 | -0.4139537 | 0.0025306 |
| CNTN4 | chr3:2629864 | -0.4203052 | 0.0021351 | TOMM5 | chr9:37590960 | -0.4139093 | 0.0025336 |
|  | chr11:306324 | -0.4201194 | 0.0021459 | PRKCH | chr14:61938004 | -0.4138857 | 0.0025352 |
| PTPRN2 | chr7:157423165 | -0.4200379 | 0.0021506 |  | chr5:268040 | -0.4136549 | 0.0025507 |

| **Supplemental Table 5 continued: DNA methylation changes as they relate to HMW adiponectin response following the anti-inflammatory nutrition supplement as assessed by Illumina (n=55)** | | | | | | | |
| --- | --- | --- | --- | --- | --- | --- | --- |
| **Gene Name** | **Chromosome Position** | **Correlation Co-efficient** | **P-Value** | **Gene Name** | **Chromosome Position** | **Correlation Co-efficient** | **P-Value** |
| **Inverse Associations** | | |  |  |  |  |  |
|  | chr3:75657183 | -0.4136444 | 0.0025514 | PTPRN2 | chr7:157643125 | -0.4089186 | 0.0028889 |
| BCL7A | chr12:122499187 | -0.413635 | 0.002552 | MICALL2 | chr7:1499391 | -0.4087979 | 0.002898 |
| WIPI2 | chr7:5263854 | -0.4135931 | 0.0025549 | SIK3 | chr11:116941490 | -0.4086633 | 0.0029082 |
| FYB | chr5:39204386 | -0.4135248 | 0.0025595 | C8orf46 | chr8:67417790 | -0.4086605 | 0.0029084 |
| C8orf34 | chr8:69665987 | -0.4134648 | 0.0025636 | RYR1 | chr19:38974350 | -0.4083942 | 0.0029287 |
|  | chr16:89098327 | -0.413387 | 0.0025689 | FCHSD2 | chr11:72724062 | -0.4081598 | 0.0029466 |
| CNST | chr1:246783166 | -0.4132458 | 0.0025785 | SDHAP2 | chr3:195384522 | -0.4081379 | 0.0029483 |
| NDRG1 | chr8:134251118 | -0.413147 | 0.0025852 | FAF2 | chr5:175875402 | -0.4078193 | 0.0029728 |
| FAM13A | chr4:89829015 | -0.4130377 | 0.0025927 | OR5E1P | chr11:7871040 | -0.4077764 | 0.0029761 |
|  | chr4:2466875 | -0.4129867 | 0.0025962 | FGD6 | chr12:95491799 | -0.4077342 | 0.0029794 |
|  | chr5:174178587 | -0.4128224 | 0.0026075 |  | chr9:125227559 | -0.4076857 | 0.0029832 |
| PAX9 | chr14:37146463 | -0.4127939 | 0.0026094 |  | chr1:201479959 | -0.407651 | 0.0029859 |
| SEC22B | chr1:145100564 | -0.4127317 | 0.0026137 | FAM128A | chr2:132250142 | -0.4074825 | 0.002999 |
| ACSF3 | chr16:89222027 | -0.4126822 | 0.0026171 |  | chr16:21568105 | -0.4074526 | 0.0030013 |
| TAOK2 | chr16:29997841 | -0.4125872 | 0.0026237 | TBL1XR1 | chr3:176916496 | -0.4074439 | 0.003002 |
|  | chr8:9742024 | -0.4124867 | 0.0026307 |  | chr1:214445695 | -0.4074283 | 0.0030032 |
|  | chr5:141742115 | -0.4121793 | 0.0026521 | CTTNBP2 | chr7:117356490 | -0.4074025 | 0.0030052 |
| KHDC1 | chr6:73973128 | -0.4120946 | 0.002658 | COL6A1 | chr21:47404216 | -0.4073889 | 0.0030063 |
| KIAA0528 | chr12:22697347 | -0.4120271 | 0.0026628 | NEDD4 | chr15:56138634 | -0.4072499 | 0.0030171 |
|  | chr6:25230306 | -0.4117855 | 0.0026797 | HMGA1 | chr6:34204698 | -0.4072222 | 0.0030193 |
| RPL27 | chr17:41150318 | -0.4115385 | 0.0026972 | UNC80 | chr2:210714233 | -0.4071104 | 0.0030281 |
| FAM24A | chr10:124670078 | -0.4114098 | 0.0027064 | MCF2L2 | chr3:182984429 | -0.4069702 | 0.0030391 |
|  | chr5:67027311 | -0.4113918 | 0.0027077 |  | chr2:43840182 | -0.4068610 | 0.0030478 |
| FSTL5 | chr4:162446260 | -0.4112551 | 0.0027174 | CENPN | chr16:81040053 | -0.4068080 | 0.0030519 |
|  | chr1:244175136 | -0.411127 | 0.0027266 |  | chr4:142253700 | -0.4068080 | 0.0030519 |
| ARL4D | chr17:41477300 | -0.4108437 | 0.0027469 | DNAH9 | chr17:11632341 | -0.4065414 | 0.0030731 |
| TMED7 | chr5:114957464 | -0.4107698 | 0.0027523 | KCNJ10 | chr1:160011013 | -0.4061781 | 0.0031022 |
| YAF2 | chr12:42632618 | -0.4107271 | 0.0027553 |  | chr4:99583945 | -0.4061023 | 0.0031083 |
| UBE4A | chr11:118230307 | -0.4104738 | 0.0027737 |  | chr16:29627881 | -0.4060146 | 0.0031153 |
| EGR2 | chr10:64576222 | -0.4103207 | 0.0027849 |  | chr1:150119278 | -0.4059264 | 0.0031224 |
| ZNF582 | chr19:56904442 | -0.4102549 | 0.0027897 | DAZL | chr3:16645732 | -0.4059234 | 0.0031227 |
| SPOCK1 | chr5:136833216 | -0.4100899 | 0.0028018 | RPS19BP1 | chr22:39928902 | -0.4058693 | 0.003127 |
| MMACHC | chr1:45965870 | -0.40984 | 0.0028201 |  | chr15:39287147 | -0.4055691 | 0.0031514 |
| IQCE | chr7:2609875 | -0.4098088 | 0.0028224 | RUNX3 | chr1:25257505 | -0.4055469 | 0.0031532 |
| MUC5B | chr11:1274269 | -0.4098009 | 0.002823 | TMC7 | chr16:19061053 | -0.4054225 | 0.0031634 |
|  | chr5:94830 | -0.4097355 | 0.0028279 |  | chr7:32904764 | -0.4052791 | 0.0031751 |
|  | chr6:150312129 | -0.4096979 | 0.0028307 | LOC387763 | chr11:43963845 | -0.4052767 | 0.0031753 |
| TCERG1L | chr10:133058601 | -0.409642 | 0.0028348 | GNB4 | chr3:179169571 | -0.4051353 | 0.0031869 |
| FAM189A1 | chr15:29432016 | -0.4095913 | 0.0028386 |  | chr8:284126 | -0.4050716 | 0.0031921 |
|  | chr5:82155751 | -0.4095188 | 0.0028439 | SCARB1 | chr12:125282561 | -0.4050469 | 0.0031942 |
|  | chr16:3155880 | -0.4091135 | 0.0028742 | MYL10 | chr7:101257675 | -0.4050001 | 0.003198 |
| CRISPLD2 | chr16:84871405 | -0.408998 | 0.0028829 | C2orf43 | chr2:21023697 | -0.4049012 | 0.0032062 |
|  | chr3:193973396 | -0.4089884 | 0.0028836 | AGK | chr7:141250424 | -0.404515 | 0.0032383 |

| **Supplemental Table 5 continued: DNA methylation changes as they relate to HMW adiponectin response following the anti-inflammatory nutrition supplement as assessed by Illumina (n=55)** | | | | | | | |
| --- | --- | --- | --- | --- | --- | --- | --- |
| **Gene Name** | **Chromosome Position** | **Correlation Co-efficient** | **P-Value** | **Gene Name** | **Chromosome Position** | **Correlation Co-efficient** | **P-Value** |
| **Inverse Associations** | | |  |  |  |  |  |
| MIR548H3 | chr6:97731400 | -0.4044498 | 0.0032437 | TCERG1L | chr10:133036065 | -0.4019987 | 0.0034542 |
| YBX1 | chr1:43152179 | -0.4040131 | 0.0032804 | RPS11 | chr19:49999222 | -0.4018545 | 0.003467 |
| ATP6V1C1 | chr8:104032860 | -0.4039197 | 0.0032883 | SND1 | chr7:127347748 | -0.4017254 | 0.0034784 |
|  | chr3:88352696 | -0.4038037 | 0.0032981 | PLCE1 | chr10:95848734 | -0.4016802 | 0.0034824 |
|  | chr4:55365203 | -0.4037057 | 0.0033064 | GPC6 | chr13:94493055 | -0.4016163 | 0.0034881 |
| DGKG | chr3:186022299 | -0.4036131 | 0.0033143 |  | chr15:74357886 | -0.4015396 | 0.003495 |
| PCDHB11 | chr5:140578896 | -0.4035839 | 0.0033167 | CXCR5 | chr11:118764003 | -0.4014789 | 0.0035004 |
| ZDHHC17 | chr12:77157699 | -0.4035341 | 0.003321 | EPHB2 | chr1:23061564 | -0.4014142 | 0.0035062 |
| CCDC134 | chr22:42197038 | -0.4034457 | 0.0033285 | HLA-H | chr6:29857512 | -0.4014097 | 0.0035066 |
| MBNL2 | chr13:97874631 | -0.4034132 | 0.0033313 | FAM84B | chr8:127566191 | -0.4013283 | 0.0035139 |
| MTRR | chr5:7869257 | -0.4033657 | 0.0033354 | OSBP2 | chr22:31298152 | -0.4011369 | 0.003531 |
| EIF2AK4 | chr15:40309387 | -0.4032666 | 0.0033439 | WDFY3 | chr4:85801119 | -0.4010943 | 0.0035349 |
|  | chr10:9920219 | -0.4032357 | 0.0033465 | RORA | chr15:61517510 | -0.4009501 | 0.0035479 |
| ZNF487 | chr10:43951555 | -0.4032067 | 0.003349 | NPHP4 | chr1:5932398 | -0.4008774 | 0.0035545 |
| MCF2L | chr13:113707517 | -0.4029931 | 0.0033674 | ROBO1 | chr3:79071297 | -0.4008455 | 0.0035574 |
| CDH13 | chr16:83659882 | -0.4029537 | 0.0033708 | FUS | chr16:31191475 | -0.4006318 | 0.0035768 |
| RARG | chr12:53614270 | -0.4029033 | 0.0033752 | ZNF747 | chr16:30546404 | -0.4006243 | 0.0035775 |
| TMPRSS11B | chr4:69093675 | -0.4028709 | 0.003378 | HAO1 | chr20:7921905 | -0.4005865 | 0.0035809 |
| OR12D3 | chr6:29342679 | -0.4028587 | 0.003379 | MUC21 | chr6:30953064 | -0.4005221 | 0.0035868 |
|  | chr2:3896439 | -0.4028295 | 0.0033816 | RPRM | chr2:154336341 | -0.4005124 | 0.0035877 |
| C7orf54 | chr7:127637871 | -0.402679 | 0.0033946 | PUS7 | chr7:105162814 | -0.4004993 | 0.0035889 |
| LHX4 | chr1:180205253 | -0.4024855 | 0.0034115 | SPDYE1 | chr7:44039612 | -0.4002262 | 0.0036139 |
|  | chr8:130389960 | -0.4024043 | 0.0034186 | INPP5D | chr2:234077733 | -0.4002107 | 0.0036153 |
| ST6GALNAC1 | chr17:74639793 | -0.402271 | 0.0034303 | NUDT22 | chr11:63993641 | -0.4001828 | 0.0036179 |
|  | chr2:20757026 | -0.4022518 | 0.0034319 |  | chr5:127909589 | -0.4000993 | 0.0036255 |
| ISLR2 | chr15:74422528 | -0.40212 | 0.0034435 | ACVR1B | chr12:52365222 | -0.4000759 | 0.0036277 |
| HAO2 | chr1:119911000 | -0.4020733 | 0.0034476 |  | chr3:75415591 | -0.4000732 | 0.003628 |
|  | chr8:143636829 | -0.4020229 | 0.0034521 | GPRC5C | chr17:72427693 | -0.4000492 | 0.0036302 |
| CUGBP2 | chr10:11059708 | -0.4019987 | 0.0034542 |  |  |  |  |
| ^1^Data analyzed by Pearson correlation analysis to examine the relationship between changes in DNA methylation and HMW adiponectin in response to the AINS (as assessed by Illumina), ^2^HMW, high-molecular-weight | | | | | | | |

| **Supplemental Table 6: EpiTYPER technical validation of DNA methylation changes as they relate to HMW adiponectin response following the anti-inflammatory nutrition supplement (n=22)** | | | | | | | |
| --- | --- | --- | --- | --- | --- | --- | --- |
| **Gene Name** | **Chromosome Position** | **Correlation Co-efficient** | **P-Value** | **Gene Name** | **Chromosome Position** | **Correlation Co-efficient** | **P-Value** |
| EGR2 | chr10:64576309 | -0.419 | 0.03 | WNT1 | chr12:49375668 | 0.027 | NS |
| EGR2 | chr10:64576288 | 0.651 | 0.002 | WNT1 | chr12:49375734 | 0.123 | NS |
| EGR2 | chr10:64576280 | -0.455 | 0.02 | WNT1 | chr12:49375745 | -0.143 | NS |
| EGR2 | chr10:64576457 | -0.151 | NS | WNT1 | chr12:49375778 | 0.004 | NS |
| EGR2 | chr10:64576422 | -0.235 | NS | INSR | chr19:7294018 | -0.432 | 0.02 |
| EGR2 | chr10:64576388 | -0.271 | NS | INSR | chr19:7293974 | 0.368 | 0.05 |
| EGR2 | chr10:64576363 | -0.272 | NS | INSR | chr19:7293969 | -0.354 | 0.05 |
| EGR2 | chr10:64576335 | -0.177 | NS | INSR | chr19:7293765 | 0.012 | NS |
| EGR2 | chr10:64576270 | -0.195 | NS | INSR | chr19:7293783 | 0.168 | NS |
| EGR2 | chr10:64576256 | -0.230 | NS | INSR | chr19:7293810 | -0.044 | NS |
| EGR2 | chr10:64576222 | 0.066 | NS | INSR | chr19:7293826 | -0.167 | NS |
| EGR2 | chr10:64576174 | -0.089 | NS | INSR | chr19:7293844 | 0.096 | NS |
| EGR2 | chr10:64576130 | -0.044 | NS | INSR | chr19:7293853 | -0.318 | NS |
| EGR2 | chr10:64576092 | 0.048 | NS | INSR | chr19:7293909 | -0.125 | NS |
| MED4 | chr13:48669166 | -0.410 | 0.03 | INSR | chr19:7294029 | 0.042 | NS |
| MED4 | chr13:48669363 | -0.405 | 0.03 | INSR | chr19:7294050 | -0.119 | NS |
| MED4 | chr13:48669356 | -0.262 | NS | KLF14 | chr7:130419254 | -0.3596 | 0.05 |
| MED4 | chr13:48669129 | -0.205 | NS | KLF14 | chr7:130419340 | -0.3056 | 0.08 |
| MED4 | chr13:48669391 | 0.022 | NS | KLF14 | chr7:130419377 | -0.1619 | NS |
| MED4 | chr13:48669329 | -0.332 | NS | KLF14 | chr7:130419370 | -0.05352 | NS |
| MED4 | chr13:48669286 | -0.193 | NS | KLF14 | chr7:130419351 | -0.1112 | NS |
| MED4 | chr13:48669280 | -0.028 | NS | KLF14 | chr7:130419236 | -0.122 | NS |
| MED4 | chr13:48669259 | 0.003 | NS | KLF14 | chr7:130419225 | 0.2391 | NS |
| MED4 | chr13:48669206 | -0.122 | NS | KLF14 | chr7:130419198 | -0.2081 | NS |
| MED4 | chr13:48669152 | -0.028 | NS | KLF14 | chr7:130419173 | -0.1871 | NS |
| MED13L | chr12:116581352 | -0.029 | NS | KLF14 | chr7:130419159 | 0.08722 | NS |
| MED13L | chr12:116581415 | 0.155 | NS | KLF14 | chr7:130419136 | 0.253 | NS |
| TBL1XR1 | chr3:176916497 | -0.243 | NS | KLF14 | chr7:130419133 | 0.253 | NS |
| WNT1 | chr12:49375609 | -0.143 | NS | KLF14 | chr7:130419118 | 0.3392 | NS |
| WNT1 | chr12:49375646 | -0.272 | NS | KLF14 | chr7:130419116 | 0.03753 | NS |
| ^1^Data analyzed by Pearson correlation analysis to examine the relationship between changes in DNA methylation and HMW adiponectin in response to the AINS (as assessed by EpiTYPER), ^2^HMW, high-molecular-weight | | | | | | | |

**Supplemental Figures**

**Supplemental Figure 1 (A) Eicosapentaenoic acid and docosahexaenoic acid as percentage of total fatty acids in cholesteryl esters and (B) Plasma α-tocopherol and lycopene concentrations post supplementation in responders (n=23) and non-responders (n=35)**

**A**

**B**

^1^Data are illustrated as mean (SEM), ^2^P-value ≤0.05 considered statistically significant as assessed by independent samples t-tests

**Supplemental Figure 2 Changes in the methylation status of CpG loci located on (A) MED4 (B) WNT1 and (C) TBL1XR1 in relation to HMW adiponectin response to the anti-inflammatory nutrition supplement as assessed by Illumina**

**A**

**B**

**C**

^1^Data analyzed by Pearson correlation analysis to examine the relationship between changes in DNA methylation and HMW adiponectin in response to the AINS (as assessed by Illumina), ^2^HMW, high-molecular-weight; FDR, false discovery rate.

**Supplemental Figure 3 Changes in the methylation status of CpG loci located on (A) INSR and (B) KLF14 in relation to HMW adiponectin response to the anti-inflammatory nutrition supplement as assessed by Illumina**

**A**

**B**

^1^Data analyzed by Pearson correlation analysis to examine the relationship between changes in DNA methylation and HMW adiponectin in response to the AINS (as assessed by Illumina), ^2^HMW, high-molecular-weight

**Supplemental Method Information**

**Exclusion Criteria**

Potential participants were excluded for any of the following reasons: known endocrine disorder, chronic inflammatory condition, kidney or liver dysfunction, iron deficiency anemia, prescribed anti-inflammatory medication, consumer of fatty acid supplements including fish oil supplements, evening primrose oil and antioxidant supplements, high consumer of oily fish (>2 servings/wk), special dietary requirements, weight change ≥3kg within the preceding 3 months, pregnancy or lactation, alcohol or drug abuse, allergy to fish and/or shellfish.

**DNA Methylation Analysis**

**DNA Extraction**

DNA was extracted from 200μl -80˚C frozen participant buffy coat (n=55) using the QIAamp DNA Blood Mini Kit (Qiagen Ltd, Crawley, UK). Buffy coats had been collected from EDTA vacutainer tubes following removal of plasma. DNA was extracted according to the manufacturer’s protocol to yield a purified sample in a rehydration volume of 400μl 1X TE (10 mM Tris-HCl pH 8.0 / 1 mM EDTA) buffer. DNA purity was assessed by 260/280 ratio, and the DNA quantified using the Nanodrop 1000 spectrophotometer (Nanodrop Technologies, Wilmington, DE).

Samples that did not meet minimum DNA concentration requirements underwent further ethanol precipitation. 40μl 3M sodium acetate (pH 5.2) was added to 400μl purified DNA in 1X TE, and mixed thoroughly. 800μl 100% ethanol was then added and the solution was stored at -20˚C for 12-18 hours. Samples were then centrifuged at 15,000rpm for 15 minutes. The supernatant was carefully decanted prior to adding 1ml 70% ethanol to the pellet. The samples were then dried in a SpeedVac and re-suspended in 35μl 1X TE buffer.

All samples had a final minimum concentration of 100 ng/μL and a minimum volume of 35 μL.

**Genome-wide DNA methylation analysis**

DNA methylation was analyzed by the McGill University and Génome Québec Innovation Centre (Montreal, Quebec, Canada) using the Infinium HumanMethylation450 BeadChip assay (Illumina, San Diego, CA, USA). Genomic DNA (500 ng) was bisulfite treated using the EZ DNA Methylation-Gold Kit (Zymo Research). Pyrosequencing was performed using PyroMarkQ24 (Qiagen) to confirm DNA quality prior to analysis of DNA methylation. The Infinium assay was conducted according to the standard Infinium HD Assay Methylation Protocol Guide.

**DNA methylation data processing**

Data was processed using the Bioconductor Minfi package in R. Raw beta values were normalized using subset quantile normalization prior to assessment of data quality. Individual probes were filtered based on Illumina detection p-values and all probes with a detection p-value >0.01 in at least one sample were removed. In total, we analyzed data across 482,596 cysteine-phosphate-guanine (CpG) loci (i.e. 99.4% of all probes). No participant samples warranted removal from the dataset. The largest percentage of failed probes within any given sample was 0.14%. Correction for batch effects within the methylation array data was performed using the COMBAT function.

Pearson correlation analysis was used to examine the relationship between changes in DNA methylation and changes in metabolic outcomes in response to the anti-inflammatory nutrition supplement. An absolute correlation co-efficient threshold of 0.4 was applied to results in an effort to determine the most biologically relevant relationships. P-values were adjusted for multiple comparisons, using the Benjamini-Hochberg false discovery rate. Adjusted p-values ≤0.05 were considered statistically significant.

The Human Reactome Network (www.reactome.org) was used for all pathway analysis. For pathway analysis, a P-value ≤0.05 was considered statistically significant as assessed by right-tailed Fisher Exact Test.

**Technical Validation**

Illumina methylation was validated with Sequenom EpiTYPER. EpiTYPER assays were designed for CpG sites which were significantly associated (p≤0.05) with HMW adiponectin response and located on genes within the adipose tissue differentiation pathway. Additionally, CpG sites located on genes implicated in T2D risk according to GWAS (McCarthy et al., 2010) which were significantly associated with HMW adiponectin change were also validated. Genomic DNA (500 ng) was bisulfite treated using the EZ DNA Methylation-Gold Kit (Zymo Research) and assays were conducted according to manufacturer’s recommendations at the McGill University and Génome Québec Innovation Centre.
